# Stakeholder perspectives on the anticipated acceptability of low-intensity psychological interventions in Java, Indonesia: A theoretically framed qualitative study

**DOI:** 10.1371/journal.pone.0358869

**Published:** 2026-09-29

**Authors:** Herni Susanti, Novitri Nurimani Asha, Penny Bee, Heni Dwi Windarwati, Budi Anna Keliat, Atik Puji Rahayu, Amy Blakemore, Afni Handayani, Mochamad Ali Sodikin, Helen Brooks

**Affiliations:** 1 Department of Mental Health Nursing, Faculty of Nursing, Universitas Indonesia, Depok, Indonesia; 2 Division of Nursing, Midwifery and Social Work, School of Health Sciences, The University of Manchester, Manchester, United Kingdom; 3 Department of Mental Health Nursing, Faculty of Health Sciences, Universitas Brawijaya, Malang, Indonesia; 4 Marzoeki Mahdi Hospital, Bogor, Indonesia; 5 Faculty of Psychology, Universitas Semarang, Semarang, Indonesia; 6 Radjiman Wediodiningrat Hospital, Malang, Indonesia; Universitas Prima Indonesia, INDONESIA

## Abstract

**Introduction:**

Depression and anxiety remain major, largely unaddressed drivers of disability in low- and middle-income countries (LMICs), where scarce resources and persistent stigma constrain access to care. Scalable solutions are urgently needed. Low-intensity psychological interventions, delivered by trained non-specialists offer a promising pathway to close the treatment gap. This study examines the prospective acceptability of these interventions in Java, Indonesia, an exemplar of an under resourced health system, to guide targeted cultural adaptation and inform optimal implementation.

**Methods:**

Using a qualitative descriptive design guided by the Theoretical Framework of Acceptability (TFA), we conducted semi-structured interviews (n = 33) with people experiencing anxiety and depression and 21 focus groups with families (n = 34), health workers (n = 36), and community/religious leaders (n = 37) across 12 sites in Java. Data were collected in Bahasa/local languages, transcribed, translated, and analysed using TFA informed Framework Analysis. 5% of translations were independently back-verified. Stakeholder workshops and advisory panels shaped study design and interpretation.

**Results:**

Low mental health literacy and stigma undermined intervention coherence and affective attitude, with many conflating LIPIs with general counselling. Help-seeking was often delayed until distress became severe, reflecting limited understanding of mental health problems and available support. Despite these challenges, strong perceptions of effectiveness and positive affective attitudes were driven by cultural values of self-reliance and community support. Concerns centered on ethicality, particularlyprivacy risks and fear of social judgment, alongside undercertainty regarding the role of traditional healers and the burden associated with time and workload demands. Trust in lay health workers was high in rural areas but more variable in urban contexts.

**Conclusion:**

Findings highlight that cultural, structural, and interpersonal factors shape anticipated intervention acceptability. While stigma and low literacy remain barriers, strong perceived need and cultural alignment suggest the potential for promising subsequent uptake. Addressing privacy, role clarity, and logistical burden through tailored adaptations and partnerships is critical. This study offers one of the first theoretically framed qualitative explorations of LIPI acceptability in Southeast Asia, highlighting culturally congruent pathways for subsequent LMIC implementation.

## Introduction

Depression and anxiety are highly prevalent mental health conditions. These conditions often co-occur, with each other and with chronic physical health conditions, substantially worsening population health outcomes [1]. The strategic importance of implementing and expanding high quality, accessible treatments for depression and anxiety is absolute. Globally, these conditions exert a high economic burden and disproportionately affect low resource countries, increasing health costs, reducing productivity and challenging sustainable development in LMIC settings [2]. Depression and anxiety are estimated to cause a global productivity loss of approximately one trillion USD annually [3]. Moreover, cognitive symptoms such as impaired concentration, memory and decision-making are common in depression and contribute substantially to reduced occupational functioning and productivity. [4]. Yet, the ratio of available mental health therapists per 100 000 population in LMICs is approximately 0·5% that of high income countries [5]. Only 7–21% of people with depression and 5–15% of people with anxiety receive minimally adequate treatment in these nations [6]. A key challenge facing LMICs, is how to provide safe, sustainable services for depression and anxiety in the community, with scarce health resources.

Effective psychological interventions exist for depression and anxiety, and are underpinned by a robust evidence base [7,8]. A global shift towards enhancing the accessibility and efficiency of mental health services has led to the development and release of ‘lower intensity’ versions of these interventions with proven effectiveness across patient groups. Popular examples include the transdiagnostic approaches integrated into stepped care initiatives in high income settings (e.g., behavioural activation, problem-solving therapies) [9] and the WHO guided self-help and Problem Management Plus programmes recommended by the WHO for low-middle income contexts [10].

Amenable to delivery by non-specialist workers, the affordability of low intensity psychological interventions is premised on the task-shifting model. The underlying assumption is that any compromise in workforce preparation will effectively be outweighed by increased service accessibility and treatment reach [11,12]. When evidence-based psychological interventions are shifted to lay workers, community mental health literacy and intervention acceptability are also believed to improve [13]. A Cochrane review demonstrates that individuals with no specialist mental health background can deliver low-intensity interventions and significantly improve depression and anxiety recovery rates in LMICs [14].

Meta-analyses have demonstrated that low-intensity interventions are effective treatments for common mental health problems and can achieve comparable effects to higher intensity psychological interventions when used in primary care settings [15]. However, the WHO is consistent in advocating that healthcare providers and decision makers should tailor task shifting models to their local demography and needs [16]. The international Programme for Improving Mental Healthcare (PRIME consortium) has long called for aggregated learning from situation analyses to strengthen emerging mental health systems [17].

Indonesia, the 4^th^ most populous nation globally, has approximately fourteen million people living with depression and anxiety, placing it in the top two countries for prevalence in the World Health Organisation’s (WHO) South-East Asia region [18]. Here, long-standing deficits in treatment access persist [19]. Recent economic growth has conferred Indonesia with middle-income status but at 2.69–3.12% of the GDP in 2022–2023, Indonesia’s total health expenditure is one of the lowest of all LMICs, and the smallest among the Association of South-east Asian Nations (ASEAN) where the regional average is around 4% [20,21]. According to the most recent basic health research survey in Indonesia, approximately 11 million people over the age of 15 years old have depression yet only 9% are receiving treatment [22]. Chronic staff shortages and professional skill mix imbalances have constrained the development of frontline mental health services and fostered an over-dependence on tertiary care [19,23].

Although WHO and PRIME initiatives have expanded scalable mental health interventions in LMICs, evidence remains limited on how socio-cultural, religious, and gender norms intersect with anticipated intervention acceptability in Muslim-majority settings like Indonesia. Robust guidance for developing and implementing large-scale primary care mental health programmes in Indonesia is lacking. While emerging evidence suggests high acceptability and satisfaction among LIPI recipients, concerns persist about feasibility, particularly the need for ongoing support and supervision for lay counsellors and implementing organizations [20,21]. To address these gaps, this study provides a theoretically framed qualitative exploration of the anticipated acceptability of a proposed low-intensity psychological intervention in Indonesia. Conducted prior to implementation, the study aimed to identify adaptation priorities, potential barriers and stakeholder perspectives to inform subsequent cultural adaptation and implementation planning. Rather than examining experienced acceptability following intervention exposure, we explore how health workers, patients, families, community leaders and traditional healers anticipated the acceptability of the proposed intervention and its delivery within their local contexts.

## Materials and methods

### Context

The study forms part of a larger programme of research, the NIHR Global Health Research Group on Sustainable Treatment for Anxiety and depression in Indonesia (Award number NIHR134638). This programme aimed to co-adapt and implement a low-intensity psychological intervention delivered by lay health workers, Kader, for adults with anxiety and depression in Java, Indonesia. The study reported here was undertaken prior to LIPI implementation, to understand cultural adaptation priorities from the perspectives of multiple stakeholders. The process of cultural adaptation is described elsewhere.

### Design

This multi-site study employed a qualitative descriptive design informed by the Theoretical Framework of Acceptability [24]. Data were collected in 12 sites across four non-special region provinces, i.e., East, West and Central Java, and Banten in Java, Indonesia. Sites span a breadth of urban, semi-urban and rural villages as defined by Indonesian National Population Census criteria.

Data were collected via focus groups and one-to-one interviews to explore the anticipated acceptability of low-intensity psychological interventions from the perspectives of people with anxiety and depression, family members health workers and community and spiritual leaders. The manuscript adheres to the COREQ guidelines for reporting of qualitative studies to support transparency [25].

This research received ethical approvals from the University of Manchester [Ref 14566], Indonesia’s National Research and Innovation Agency [Ref: 010/KE.03/SK/11/2022] and the Faculty of Nursing, the University of Indonesia [Ref KET-237/UN2.F12.D1.2.1/PPM.00.02/2022.] The study followed ethical research principles based on the Belmont Report which includes the principles of beneficence, justice, and respecting human dignity. Written consent was obtained from all participants prior to participation.

### Community engagement and involvement (CEI)

Study design: We facilitated a 3-day workshop with 22 high influence Indonesian stakeholders with the intention of identifying the contextual issues challenging implementation. We also met with 24 representatives from primary care services across Java and ran two Community Engagement and Involvement (CEI) consultation meetings to further identify and incorporate local challenges into our study design.

Project management: We partnered with four Indonesian mental health community groups who established an independent and diverse CEI advisory panel (n = 8) and convened a complementary lived experience advisory panel (n = 9) to support study activities. These advisory panels provided inputs into recruitment pathways, participant facing study documentation and interviews/topic guides. One member of the lived experience panel also joined the authorship team and contributed to the analysis of transcripts and development of the manuscript. We translated, culturally adapted and delivered a research methods training course for public and patient contributors and delivered this at study outset to support involvement throughout the study [26].

### Sampling and recruitment

Participants were purposively sampled based on the criteria outlined below to ensure diversity in terms of geographical area, gender, age and diagnosis. Data collection commenced until data saturation was achieved and within the parameters of the sample size agreed with study funders (20–25 per stakeholder group).

Inclusion criteria:

Participants must be aged 18 years or over and be one of the following: Health workers with current or future responsibility for delivering mental healthcare. People with experience of anxiety and/or depression within the last 12 months, irrespective of engagement with mental health services. Family members of individuals who have experienced anxiety and/or depression within the last 12 months, irrespective of engagement with mental health services. Third-sector professionals or community stakeholders with current or potential future experience of supporting people with anxiety and/or depression in the study sites.

Exclusion criteria:

- Those with recent bereavement, postnatal depression or those who meet a screen for recent or current suicide risk.

Patients and family members were recruited through primary care services and other relevant health and community settings. The study did not aim to establish clinical diagnoses or compare diagnostic groups. Instead, participants were recruited based on lived experience of anxiety and depression within the previous 12 months, consistent with the exploratory aims of the study and its focus on stakeholder perspectives regarding the anticipated acceptability of the proposed intervention. Individuals with lived experience were identified through primary care and community networks rather than formal diagnostic procedures, reflecting the intended community-based implementation context of the intervention. Health and community workers distributed study information packs, including an invitation letter, participant information sheet and consent-to-contact form with return envelope, on behalf of the study team by post or email.

Other stakeholder groups were invited through direct invitation from the study team. Invitation packs containing an invitation letter, participant information sheet and consent to contact form (either by post, phone or digitally via email) to professional contacts known to the study team through links to 12 study sites such as professionals, lay workers, spiritual/traditional healers/ community leaders, government ministers, policy makers etc. Participants contacted the study team directly if they were interested in taking part.

Researchers made contact with interested parties and arranged a convenient date for focus group/and or interview.

### Data collection

Following consultation with local collaborators and given the sensitive nature of the interview topics, semi-structured interviews were conducted with people with lived experience of anxiety and depression [27] (n = 33). The remaining participant groups took part in focus group discussions (n = 21), enabling interrogation of both individual and collective views. During early data collection activities, researchers identified some uncertainty regarding the nature and purpose of low-intensity psychological interventions. In response, a standardised description of the proposed intervention, including its purpose, format, duration and delivery model, was provided at the beginning of subsequent interviews and focus groups to support participant understanding and facilitate meaningful discussion.

Data was collected between March and August 2023. On average, semi-structured interview lasted between 30 and 70 minutes while focus group discussions last just between 60 and 70 minutes. Interviews were conducted in-person and at either participants’ homes or accessible community venues based on participant preferences and individual circumstances. Focus groups were held at local community venues, contained five participants per focus group to optimise discussion and facilitated by a minimum of two researchers. Data was collected in either Bahasa Indonesian (national language) or local languages (Javanese and Sundanese) depending on participant preference at each site. Interviews and focus group were recorded using digitally encrypted recorders and uploaded to secure servers at Universitas Indonesia as soon as possible after data collection. Recordings were then transcribed verbatim into the language in which it was undertaken prior to being translated into English for purposes of analysis. Original transcripts were retained to support the analysis process. As recommended, 5% of transcripts were back translated into the original language by an independent UK based translation company to ensure accuracy [28].

### Participants

140 participants were recruited in total exceeding initial sample estimates. These including 33 people with anxiety and depression, 34 family members, 36 health workers (including professional and lay health workers) 37 community, religious and spiritual leaders. See Tables 1–2 for more details on study participants.

**Table 1:** Breakdown of participants across sites and groups.

Province	Site	People with anxiety and depression	Family members	Community leader, spiritual leader, and Kader	Health workers(GP, Nurses)	Total
Banten	**Tangerang** **city**					
	Cipadu	5	5	5	5	20
West Java	**Bogor** **District**					
	Gn Bunder 1	3	3	4	3	13
	Gn. Bunder 2	4	3	3	4	14
	**Bogor** **District**					
	Cibunian	3	3	3	3	12
Central Java	**Magelang** **District**					
	Borobudur	2	2	3	3	10
	Mungkid	2	3	2	2	9
	**Semarang City**					
	Bandarharjo	2	3	4	5	14
	Bululor	3	2	1	0	6
East Java	**Jombang** **District**					
	Kabuh	3	2	2	2	9
	Dukuh Klopo	2	3	4	4	13
	**Malang District**					
	Bantur	0	3	3	3	9
	Bululawang	4	2	3	2	11
	**Total**	**33**	**34**	**37**	**36**	**140**

**Table 2:** Participant demographics.

Stakeholder group	% (n)
***Patients (n = 33)*** Gender Male Female Age Mean Range	18.2% (6) 81.8% (27) 37.42 years 18-75 years
***Family members (n = 34)*** Gender Male Female Age Mean Range Who do you provide care for? Spouse/partner Parent Son/daughter aged over 18 Son/daughter aged under 18 Sibling Other	32.4 (11) 67.6 (23) 46.88 years 26-69 years 26.5 (9) 5.9 (2) 38.2 (13) 2.9 (1) 17.6 (6) 8.8 (3)
***Health workers (n = 36)*** Gender Male Female Age Mean Range Role Lay health worker Clinician	25 (9) 75 (27) 40.61 25-59 8.3 (3) 91.7 (33)
***Community/spiritual/traditional healer or leader (n = 37)*** Gender Male Female Age Mean Range Role Community leader Faith/religious leaders Traditional/alternative/complementary healer Other	48.6 (18) 51.4 (19) 46.19 23-60 51.3 (19) 16.2 (6) 29.7 (11) 2.7 (1)

### People with anxiety and depression

Most participants were female, aged between 20–75 years. The majority of patients had accessed some form of mental health service, ranging from Community Health Center (PHC), mental health hospitals, traditional healers, religious communities, and Kader. However, a few patients reported never accessing any mental health care at all. Around half of the patients had completed only elementary or middle school, while the rest had reached high school or bachelor-level education.

#### Family members.

Most family members were female and aged between 28 and 63 years. Family roles varied, including parents, siblings, spouses, nieces, and friends. The length of time they had cared for a family member with anxiety and depression ranged from 6 months to five years.

Most caregivers had an educational level of elementary or middle school, and only a few had completed high school or higher education. Family members reported previously accessing a wide range of mental health services, including public health centers, mental health hospitals, Kader, and religious or traditional healers, often relying on community networks such as friends and spiritual leaders.

#### Health workers.

Most professional health workers were female with ages ranging between 25 and 59 years. Their roles varied, including general practitioners, nurses, midwives, pharmacists, mental health program staff, and heads of community health centers. Length of experience of working with people with anxiety and depression ranged from 6 months to 30 years with the majority having experience of five years of more.

Lay health workers or ‘kader’ were mostly female and were between 30 and 59 years old. They served either as mental health kader specifically or as kader in non-mental health areas such as general health promotion. The majority had been working in their roles for more than five years, with a few newer to the role (between 6 months to 2 years). Educational backgrounds among kader varied. Most had completed high school or middle school, while a few held diploma-level qualifications. These characteristics align with the experiences of mental health Kader supporting people with schizophrenia in Indonesian community services, where the majority were married women balancing family obligations with their volunteer roles [29,30].

#### Community, religious and spiritual leaders.

These participants were mostly male and were between 23 and 60 years old. The majority of participants identified as traditional healers or religious community leaders, with long-standing involvement in their communities — most having served in their roles for over five years. Their roles included spiritual support, healing rituals, and community guidance. Educational attainment was generally low among this group, with most having completed only elementary or high school.

### Data analysis

Data were analysed using Framework Analysis [31] informed by the Theoretical Framework of Acceptability (TFA). The TFA defines acceptability across seven components: affective attitude, burden, ethicality, intervention coherence, opportunity costs, perceived effectiveness and self-efficacy providing a conceptual scaffold to analyse data. Using the TFA prior to implementation also supported the development of recommendations to inform the cultural adaptation of our candidate intervention.

The first stage of the analysis involved familiarisation with the data. Researchers were allocated transcripts to read and then met to operationalise TFA constructs in relation to our study data and cultural context. This noting of where components might appear in the data and subsequent discussions led to some refining of TFA components (e.g., specifying collective and individual ethicality and and agreeing to include the perceived need for the intervention within the affective attitude component) prior to moving to the second stage of analysis. Indexing was then undertaken whereby our pre-agreed TFA codes were applied to the segments of text within transcripts that they applied to directly into a Word document. Next data were charted into an Excel sheet where data from each transcript were summarised in relation to TFA codes. Additional columns were included to allow for the inclusion of data that fell out with the TFA framework. Each stakeholder group was allocated a unique sheet on excel to support the contrasting of perceptions across stakeholder groups (See S1 Appendix for cross-stakeholder similarities and differences). The final stage involved the interpretation of overarching themes from the charted data recommended to capture nuance and context-specific insights into the overall perceived acceptability of low-intensity psychological interventions in Indonesia. Key questions were asked of the data at this stage including: relationships between constructs, differences within and between stakeholder groups, where does acceptability break down, what are the most salient constructs within the data?

### Reflexivity

In order to ensure reflexivity, an integral feature of qualitative analysis, during data collection and analysis the dynamic interaction between researchers, emerging analysis and participants were continually reviewed and discussed amongst the research team supported by the use of reflexive memos.

The research was predicated on the potential value of low-intensity psychological interventions for people with anxiety and depression. The research team reported no additional predefined expectations relating to data analysis that might have impacted on the research process. The research team included a wide range of experience including clinical, academic and lived experience. Seven members of the team were from Indonesia including a member from the lived experience advisory group. Interpretations and emerging analysis were discussed and agreed within this team to enhance the credibility of analysis and ensure analysis remained grounded and contextually sensitive. There were no reported existing relationships between researchers and study participants, and an audit trail was kept of indexing and charting stages to ensure transparency.

## Results

### Lack of adequate mental health literacy as a threat to anticipated intervention acceptability

Across the 12 study sites, most participants, particularly non-professionals, had limited awareness of mental health which undermined potential ***intervention coherence***. Specific diagnoses including anxiety and depression were rarely utilised and instead participants referred to participants as either “orang dengan gangguan jiwa” (people with mental health disorders) to reflect more serious mental illnesses such as psychosis or “orang dengan masalah kejiwaan” (people with psychological problem who are risk of developing mental health disorders). Help seeking was generally only undertaken once distress became severe or physical symptoms had manifest (e.g., persistent stomach pain). As a result, help-seeking behaviour for anxiety or depression was rarely reported across transcripts indicating challenges to ***perceived effectiveness*** and ***affective attitude***.

*“’So far, there are a lot of people in us who are not already aware, right, but they don’t know what the level of depression is, they think it’s normal, because they don’t know which one is normal”* – *(FGD, 6HW1, Nurse, Urban, Gunung Bunder1, Bogor)*

*“Because in reality, people come to the puskesmas for things like coughs and colds. If they are anxious, they don’t come for a check‑up, so their mental health issues are not examined. In this community, people prioritize small physical wounds over psychological wounds. What we want is to be given promoters or facilitators, so there are classes that make us more aware of these issues. Meanwhile, the government, the government’s program is only for Mental Health staff. My hope is that all staff can also be given education*.” *(FGD, 8HW5, Nurse, Rural, Cibunian, Bogor)*

*“in the past, I sought help by doing islamic spiritual healing practice because my parents did not understand about mental health and I did not understand either. “(IDI, 1P4, Patient, Urban, Tangerang)*

Due to the limited awareness of mental health and non-pharmacological interventions, there was persistent ***low intervention coherence*** across stakeholder groups in terms of content, aims and underlying mechanisms. Whilst researchers provided an overview of the intervention, participants commonly conflated LIPIs with general counselling services rather than aligning them with principles of guided self-help. This is likely due to their familiarity with school- or clinic-based counselling, which represents the most accessible and known form of mental health care in Indonesia.

“*In Indonesia we haven't, or there is, or I’ve never heard of it before... I really want it, e… I really agree… like for example, When my child and the others met, then they talk. like that..” (FGD, Family, IF2, Urban, Tangerang)*

“*Maybe it can; it can be accepted in society. Yesterday there was counseling for older people; I think that’s good. asking ‘whether you are worried or not? Then if it’s hard to sleep at night or not’, that’s good, maybe ma'am*” *(IDI, 5P5, Patient, Dukuhklopo, Jombang, Urban)*

Participants, particularly health workers and spiritual, traditional and religious leaders highlighted the difficulty of outreach and identifying participants who would be eligible for such an intervention due to this lack of awareness. This was further compounded by high levels of community stigma relating to mental health negatively impact on ***affective attitude***. Even when someone was identified, engaging them in meaningful conversations about mental health was considered to likely require a substantial ***perceived burden*** for these groups of stakeholders which limited potential subsequent acceptability.

*“So, most of them — well, those with severe mental illness don’t really understand, but those with anxiety usually don’t want anyone to know. They try to look happy, but when we ask or touch on the topic a little, everything just pours out. That’s usually what happens, Ma’am… (FGD, 1HW4, Nurse, Urban Tangerang)*

### Strength of perceived need and alignment with individual and cultural values as drivers for anticipated acceptability

Participants across stakeholder groups felt strongly about the need for the intervention in Indonesia (***affective attitude***). The strength of this view appeared to be fuelled by the lack of alternative provision for those with anxiety and depression. This gap reinformed views about ***perceived effectiveness*** and were particularly salient amongst participants in rural areas and amongst health care workers.

“*In those areas, people may face financial or social barriers. Individuals with mental health problems, or those who are angry or emotionally unstable, often feel offended or marginalized if they are told they need therapy. They may think, ‘I’m fine, getting angry is normal, it’s just human emotion.’ That’s why we need other techniques, different approaches, or additional input that we can understand and apply, so we can reach those areas that are not currently reached by medical services*” *(FGD, 2TH3, Religious Leader, Mungkid, Rural)*

“*In Indonesia we haven't, or there is, or I’ve never heard of it before... I really want it, e… I really agree… like for example, When my child and the others met, then they talk.. like that..Because probably when he’s with his mother he can’t open up, maybe if we can talk like this maybe he can open up, like that… maybe it’s like that, like me, you too, who have children like this also want to know what kind of attitude that we can take… that’s what I probably think, I think I've done my best.. but it turns out that for this child there is still something missing, you know… or something is wrong, or anything…” (FGD, IF3, Family, Tangerang, Urban)*

“*But it’s [LIPIS] actually very, very important. The real problem because we don’t have the manpower. So those who are specifically for listening, especially for counseling, because they don’t exist yet, so actually the community’s need for it, if it’s available, we can socialize it and we can convey it.” (FGD, 9HW1, Head of Puskesmas, Bandarharjo, Semarang)*

Across stakeholder groups, there was a shared view that the intervention could potentially be suitable for most people in Indonesia but may have particular relevance to those who experience limited access to psychosocial support or formal mental health services. Participants consistently emphasized the appropriateness of the intervention for people facing social isolation, limited mobility, or restricted economic means illustrating potential ***ethicality*** alignment. The modality of a guided self-help booklet was perceived to empower these individuals by promoting self-reliance and autonomy in a culturally acceptable manner.

*“I think it is really very much needed. Because we need to be aware of ourselves, about what we feel, about our mental health. For me, I think it becomes important to learn about those things.” (IDI, 1P5, Patient, Urban, Tangerang)*

*“Because of debt, because of economic problems, because they cannot afford food. With services like this, people’s minds can be opened. They can think, ‘Oh, I should do this or that.’ Then people like that won’t feel hopeless, won’t be stuck thinking they have no options. They can be guided in their work—whether to take part‑time jobs or something else—because usually people’s thoughts go in many different directions.” (IDI, 10P3, Patient, Bululor, Semarang)*

*“For someone like me who doesn’t have many friends—I rarely socialize. When I’m at work, I have many friends, but outside of that, not so much. So I think counseling is most suitable for children who don’t have many friends.” (IDI, 5P4, Patient, Dukuhklopo, Jombang)*

In terms of participants’ emotional response or ***affective attitude*** to the intervention, there was a general sense of hope and optimism across stakeholder groups about the potential for ***perceived effectiveness*** of these interventions in bringing about positive changes to themselves or the people they cared for demonstrating potentially strong alignment with individual ***ethicality***. Family members, in particular, highlighted the potential for such interventions to reduce their own emotional and caregiving ***burden***. High levels of perceived readiness to engage were evident across stakeholder groups, not only in their motivation but also in their willingness to absorb the perceived ***opportunity costs*** of participating (e.g., time and disruption to existing formal and informal provision).

*“Because with that, it can help the family. Because if our child has been cared for in such a way, we don’t want anything to be helped, and the peacefulness, in the house will get calm...”“That’s why if there is a solution for special care for children like this, yes, I am very grateful, I really want it, this is exactly what we hope for...” (FGD, 1F3, Family Member, Urban, Tangerang)*

*“So if, for example, someone has just started experiencing a mental disorder, it’s actually easy for us. We can just go there, ask for a consultation, see how it works—no need to go all the way to the psychiatric hospital. Because for people here, if you say ‘go to the hospital,’ they get worried and don’t know what to do..” (FGD, 3F2, Family, Borobudur, Magelang, Urban)*

Participants emphasized that, to fully realise intervention benefits, LIPIs must be adapted for the local context to ensure cultural relevance, reflecting considerations of alignment with values (***ethicality***). They also stressed the need for clear articulation of intervention purpose and process to potential participants and families, which is critical for ***intervention coherence*** and ***affective attitude***, supporting the potential of subsequent uptake and engagement.

“it’s just that the way of delivery, the way, the way of distribution to the house, to the community... it has to adjust to the place, that’s all” *(FGD, 11HW4, Nurse, Rural, Bantur, Malang)*

Spiritual and traditional healers and community and religious leaders also expressed positive ***affective attitudes*** toward the intervention. Many welcomed the program as a ***potentially effective*** alternative for addressing psychological distress, particularly when it was framed in alignment with religious and communal values (***ethicality***). Although few participants had a full understanding of how the intervention worked, some viewed its compatibility with Islamic principles, including community connectedness and a holistic view of health, as a key strength. This enthusiasm, although tempered by concerns about burden, suggests that traditional healers could serve as important allies in promoting the intervention, particularly if their roles are respected and meaningfully integrated into its delivery..

“*We do really hope that someday, there would be a program that can include all people in the community we can take part in this matter because mental issues can be dangerous as other kinds of illness. I believe that by involving the community in addressing mental health issues, we can achieve a better quality of life. As Muslims had been taught that being healthy means not only being physically fit but also having a healthy mind. So, when everyone can be involved in this matter, they may also be more responsible for mental health as well.* “ *(FGD, 3TH2, Traditional Healer, Urban, Borobudur, Magelang)*

### Stigma, privacy and the social risks of engaging with low-intensity psychological interventions

Pervasive individual, family and community-level stigma related to mental health were dominant within transcripts across stakeholder groups which reflected potential ***ethicality*** concerns about LIPIs alignment with social norms. People with anxiety and their families described how fear around being labelled ‘mentally-ill’ would deter both individuals and families from seeking help and limited anticipated acceptability and potential ***perceived effectiveness*** of any community outreach for the intervention.

“*Actually, I’m the one who feels embarrassed, Miss. I’m normal, so why do people say I’m mentally ill? My husband never mocks me like that, never. It’s just me who feels ashamed — I’m normal, but people say I’m mentally ill.”(IDI, 4P3, Patient, Rural, Kabuh, Jombang)*

Health workers and Kader shared similar concerns, noting that the repeated visits necessary for any subsequent implementation might unintentionally expose participants to scrutiny:

*"That’s the obstacle, yes. When it comes to anxiety and depression, when we invite them to go there, they still feel embarrassed. They don’t want to. They’re afraid people will say they have a mental disorder." (FGD, 5HW6, Health Worker, Urban, Dukuhklopo, Jombang)“"Because they think that... going to a psychologist means they’re considered mentally ill, like a crazy person. But actually, depression isn’t the same as being crazy, right?*" *(FGD, 6TH1, Kader Urban, Gunung Bunder 1, Bogor)*

Despite the potential ***ethicality*** alignment described in the previous section, the analysis also revealed anticipated tensions between the intervention and cultural values relating to privacy, social respectability and emotional restraint. Feelings of shame and fear of social judgement reflected concerns about preserving personal dignity, while the perceived need to maintain family and community reputation was viewed as a barrier to engagement and a factor likely to undermine the intervention’s effectiveness.

*“Oh, if it’s workers from puskesmas, I guess it can still be done, but from what you said earlier, the kader, it means that our people are also involved in the community, like posyandu’s kader, that’s the term, right? So no, I don’t trust them.” (IDI, 1P6, Patient, Urban, Tangerang)*

“*The problem is that many people can’t be good listeners. they can’t keep secrets or they rush to give advice, so they don’t take the time to really listen. Instead, they respond with things like, ‘You shouldn’t be like that,’ and that makes the person who wanted to share their feelings stop talking and think, ‘Why am I being blamed instead?’”(FGD, 3HW1, Nurses, Rural, Mungkid, Magelang)*

Participants emphasized that the success of the intervention depended heavily on delivering it in ways that protect privacy and ensured psychological safety (anticipated ***ethicality*** alignment). Both individual and collective concerns such as avoiding intimidation, respecting personal boundaries, and aligning with social norms around discretion appeared to strongly influence how acceptable, safe and effective the intervention was anticipated to be. These cultural expectations and perceived stigma underpinned antiicipated ***self-efficacy*** to engage with the intervention.

*“Yes, because when people share their stories, they shouldn’t have to defend themselves. The problem is that most people give advice in a condescending manner, which makes me feel belittled.” (IDI, 7P1, Patient, Rural, Bogor)*

*“Well, whether male or female, the most important thing is that there is no coercion. Because if someone is forced, it doesn’t feel good, it doesn’t come from within themselves. If there is coercion, it becomes unpleasant—some may feel fine, but others may become lazy or unwilling.” (FGD, 12F4, Family, Urban, Bululawang, Malang)*

### The influence of trust, role clarity and perceived burden in shaping anticipated acceptability

In order to overcome these barriers to individual motivation, participants described the need for external validation and support from trusted community members such as health professionals or other individuals with community standing. This included a need for social reassurance and community guidance to navigate the complex social dynamics associated with mental illness in Indonesia (***ethicality***) to support subsequent engagement with the intervention. Such validation was perceived to have the potential to play a crucial role in reinforcing individual ***self-efficacy*** to engage in the intervention latterly especially in cultural contexts where engagement is limited by uncertainty and stigma.

Kader-led delivery was pre-established and formed a core component of the intervention model. This was mostly supported in the data across stakeholder groups due to existing high levels of trust in Kader with participants describing them as approachable and credible figures within the community (***ethicality*** alignment). Empathetic and non-intimidating communication was considered essential for successful engagement, and Kader were generally viewed as capable of providing this (***self-efficacy***). Such perceptions were particularly salient among family members and those from more rural areas.

Those from more urban areas were more sceptical about the trustworthiness of Kader in delivering psychological interventions. This was due to concerns around gossip, threats to confidentiality, and the sufficiency of Kader skills and qualifications to deliver appropriate support for anxiety and depression (**self-efficacy**). Empathy, professionalism, and confidentiality were critical to participant confidence and for some participants this meant they viewed this as a threat to ***perceived effectiveness*** and would prefer that trained health workers delivered interventions for mental health.

*“I guess it’s possible, but not everyone would want to share much, probably just the important points, because people also know that these are the Kader chosen by the old people. What if they gossip? Some will fear it happening.” (1P6, Urban Area, Service User, Urban, Tangerang)*

There were confusion and uncertainty amongst spiritual and traditional healers about what their role would be in the subsequent implementation of the low-intensity psychological intervention within communities. These participants were less familiar with such interventions and as such were unclear on their benefits and relevance to their usual healing methods (potential ***ethicality*** misalignment).

*“yes, spiritual method. I think it is enough. Rather than we give them books, I am afraid the books won’t be read, books give them more headache. They already had enough headache from their life problems “ (FGD, 1TH2, Spiritual Healer, Urban, Tangerang)*

Various concerns about the potential ***burden*** and ***opportunity costs*** of intervention involvement were identified across transcripts and are likely to impact the anticipated acceptability of the intervention. This included time and logistical concerns for both patients and Kader due to the multiple session delivery approach. Kader’ existing workloads and concerns about additional strain threatened contextual integration, and patients—particularly those from low-income families—struggled to envisage how such sessions would align with their existing employment or caring responsibilities (***ethicality)***.

*Doctor, we can’t deny it the kaders (community health volunteers) are already giving their time while having many other responsibilities, and the incentives they receive from the related parties are still very minimal. So, we feel limited in asking them to help with A, B, or C, because there’s no proper compensation, and it feels uncomfortable.”(FGD, 2HW4, Nurse, Rural, Magelang)*

*“Well, because there are many grandchildren, there are also many needs, and their income is limited. No, just find the right time to confide.” IDI, 6P2, Patient, Urban, Gunung Bunder 1, Bogor*

The financial implications of the intervention also presented both potential benefits and burdens, influencing participants’ perceived ***opportunity costs***. The proximity of Kader and community health centres made the cost of treatment relatively low, as it reduced travel expenses and time away from work or family duties, aligning with values of convenience and affordability (***ethicality***). However, additional anticipated ***opportunity costs*** were noted, such as the cultural expectation to provide food or snacks during sessions, which though modest represented a trade-off against limited household budgets, potentially diverting funds from other essential needs. Women reported challenges incorporating the intervention on top of existing household responsibilities and needing permission from husbands limiting potential ***self-efficacy*** to participate.

*“My job starts at night and I rest during the day, so it’s different from the Kader’s worktime.”(IDI, 1P3, Patient, Urban, Tangerang)*

*“Busy taking care of children, busy having to take care of the husband and children, not receiving permission from husband.”(IDI, 7P4, Patient, Rural, Bogor)*

*“If everything is borne by the Kader, it’s a shame—they also have families.”(FGD, 5HW2, Kader, Urban, Jombang)*

For those that would predominately deliver the intervention, Kader and supervising nurses, the ***burden*** was anticipated to be largely practical and structural. Many Kader were already tasked with delivering other public health programs, and there was a concern that the new intervention would be added to their workload without additional resources or dedicated staff. Nurses echoed these concerns, noting that the intervention, while potentially beneficial, would require additional supervision and training time, which would be difficult to sustain given staff shortages and broader service demands. These operational challenges underscored the anticipated ***burden*** of implementing the intervention within existing health system constraints.

Suggestions to address such concerns included:

- Providing standard operating procedures or step-by-step guidance for Kader to support delivery.
- Offering additional support to other stakeholder groups such as information leaflets to increase awareness of anxiety, depression, and low-intensity interventions, alongside clarity about each group’s role in the process.
- Onboarding meetings could further support role clarity and ensure buy-in for subsequent implementation.

## Discussion

This study aimed to explore the anticipated acceptability of low-intensity psychological interventions in Java, Indonesia, drawing on perspectives from service users, health professionals, Kader who were expected to play a central role in future delivery, traditional healers and community leaders across twelve diverse implementation sites. While there was broad consensus on the urgent need for such interventions for people with anxiety and depression in Indonesia and broad cultural alignment, a number of threats to subsequent uptake and engagement were identified. These included low mental health literacy within communities, high levels of stigma, cultural misalignment in relation to values around privacy and emotional restraint, practical and financial burdens, and concerns about role clarity and anticipated implementation feasibility. However, high levels of trust in community health workers (Kader) in rural areas, emotional receptivity to the intervention, and alignment with cultural values of autonomy and communal support were perceived to be key drivers of anticipated intervention acceptability.

Low levels of awareness about mental health, specifically anxiety and depression, and non-pharmacological interventions was persistent across transcripts which broadly reflects literature from other low resource settings [32]. This low mental health literacy coupled with the novelty of the proposed intervention resulted in low intervention coherence across stakeholder groups [24]. Specifically, guided self-help was conflated with counselling meaning there were potential areas of misalignment in terms of expectations around levels of input from the person delivering the intervention, the level of individual input, intensity and duration and level of individualisation. Such misalignments have been identified in high-income settings as key barriers to intervention engagement [33]. This suggests a need for more targeted and contextualised education around mental health and intervention aims tailored for stakeholder groups to align expectations prior to subsequent use. Whilst limited intervention coherence constrains interpretation of some acceptability domains, it also demonstrates the value of undertaking prospective acceptability work prior to implementation, as these misunderstandings may not otherwise have been identified until intervention delivery had already commenced. The findings therefore highlight important areas requiring clarification during subsequent adaptation, training and implementation activities.

Despite low levels of mental health literacy and limited intervention coherence among stakeholders, a strong tension for change, driven by perceived need, was a powerful motivator that appeared to override these barriers, fostering a perceived need for alternative approaches across all groups. The perceived lack of alternative services for anxiety and depression, alongside concerns regarding the progression of these conditions into more severe mental health disorders if not treated appropriately generated a clear demand for alternative approaches. The guided self-help format was perceived as empowering and particularly relevant in the Indonesian context given identified preferences for privacy and autonomy and because they were considered to have the potential to address existing health inequalities affecting individuals with limited mobility, economic constraints, or caregiving responsibilities. This resonated strongly with existing evidence that such interventions can offer a scalable and culturally appropriate alternative to more resource-intensive interventions in low resource contexts [34].

Affective attitude, how stakeholders feel about an intervention, is a critical determinant of acceptability, influencing intervention acceptance and engagement irrespective of the strength of the underlying evidence base [35]. In this study, participants expressed positive emotions such as hope, optimism, and acceptance toward the low-intensity intervention, despite the initial limited understanding. These responses appeared to be strongly shaped by anticipated alignment with cultural values, including autonomy, self-reliance, and community support. This suggests a novel pathway through which affective attitudes may be strengthened: by aligning intervention framing with culturally embedded norms and beliefs [36,37]. Spiritual and traditional healers similarly welcomed the intervention if it could resonate with religious and communal principles, such as Islamic teachings. However, role ambiguity, particularly among religious and community leaders, was a threat to anticipated acceptability and highlighted the need for tailored education and communication strategies to clarify the intervention’s purpose and define stakeholder roles [38]. Addressing this ambiguity is likely to further strengthen affective attitudes and support subsequent implementation.

Within our data the cultural context in Indonesia, significantly shaped perceptions of anticipated acceptability. Existing trust in Kader was a key facilitator to acceptability especially in rural areas. However, those from more urban areas were more likely to be concerned about the potential ability of Kader to maintain confidentiality. Concerns about social judgement has been highlighted previously as barriers to help-seeking so any subsequent use of low-intensity interventions in Indonesia would need to consider carefully how to address such scepticism amongst potential intervention recipients [39]. These views were reflective of broader community stigma related to mental health which was considered to potentially be detrimental to later engagement with and uptake of our intervention [40]. Our findings extend current understanding by highlighting how the modality of the intervention (e.g., multiple home visits) not just the content may conflict with norms of discretion even when trust in providers was high. These findings point to the need to concomitantly deliver bespoke community-based awareness raising to reduce stigma and promote help-seeking. Partnering with trusted community figures, such as spiritual and religious leaders to deliver this could potentially help to the credibility and cultural sensitivity of such approaches [41].

Participants’ perceptions of burden varied, with some finding the proposed activities manageable and minimally disruptive, while others cited significant operational and practical barriers, such as time constraints or difficulty understanding the material. Kader and nurses, central to later delivery efforts, foregrounded concerns about workload, limited staffing and a lack of sufficient resources to deliver the intervention. Such concerns about structural barriers have the potential to threaten subsequent implementation and support the wider literature relating to task shifting approaches in global mental health [42]. Our findings go beyond logistical and structural barriers and revealed how gendered caregiving roles and household power structures further influenced anticipated burden and feasibility. For people with anxiety and depression, especially those from low-income households, time constraints, the existing burden of household responsibilities and needing permission to participate from family members limited their confidence in being able to subsequently engage with the intervention. Although most participants coalesced in their perception of the intervention being potentially low cost, other costs such as the cultural expectation to provide snacks during visits – represented a meaningful anticipated burden to those included in the studies. Importantly, a key aspect of our findings is the clear emphasis on the necessity of involving diverse local stakeholders—including traditional healers, health workers, and community leaders—in the tailoring of implementation strategies. This participatory approach was identified as critical to ensuring cultural sensitivity, enhancing acceptability, and supporting sustainable integration of the intervention within complex community contexts [43].

Across domains, anticipated acceptability appeared less as an individual cognitive judgement and more as a socially negotiated process shaped by trust, privacy, family relationships, community legitimacy and cultural expectations. This suggests that the acceptability of psychological interventions in LMIC settings may be understood not solely through individual perceptions of intervention attributes but also through the social and relational contexts in which intervention decisions are made.

Although the TFA provided a useful structure for assessing anticipated acceptability of the proposed intervention, the purpose of this study was not to generate a new theory of acceptability. Rather, the TFA was used as a sensitising framework to understand stakeholder perspectives and identify adaptation priorities prior to implementation. Nevertheless, our findings suggest that acceptability in this context may be understood as a socially negotiated process shaped by trust, privacy, family relationships, community legitimacy and cultural norms. It is also worth noting that the TFA development was based on an overview of systematic reviews largely dominated by high-income country literature, meaning that conceptualisations of acceptability from low- and middle-income countries (LMICs) were likely underrepresented [24]. This raises broader questions about whether these core domains of acceptability, and their relative weighting, transcend cultural and delivery contexts. Constructs such as opportunity costs, ethicality, or burden may hold greater or lesser significance depending on sociocultural norms, household decision-making dynamics, and resource constraints. Indeed, subsequent studies conducted in LMICs such as Fiji, Kenya and Zimbabwe have extended the TFA by adding dimensions such as unintended negative consequences, perceived social acceptability, and contextual relevance to better capture culturally shaped expectations and collective forms of participation [44–46]. While our pre-implementation study did not identify negative consequences, this may reflect the limited intervention coherence and timing rather than their absence. Consistent with prior LMIC work, our findings highlight the heightened salience of burden and costs in low-resource settings, where even modest anticipated financial or time demands can meaningfully influence acceptability [44]. During the familiarisation stage of analysis, we also found it important to distinguish between individual and collective efficacy, supporting proposals to incorporate perceived social acceptability as a separate construct in contexts where decisions are made jointly within families or communities [47]. Finally, our analysis suggests that perceived need may warrant recognition as its own component of anticipated acceptability, particularly in LMIC settings where mental health literacy, cultural interpretations of distress, and competing livelihood priorities strongly shape whether people believe they require help in the first place. This omission risks overlooking a key determinant of help-seeking and, ultimately, whether an intervention is viewed as potentially necessary or valuable in certain cultural contexts.

### Strengths and limitations

The study draws its strengths from the combination of in-depth qualitative interviews and focus groups and the use of an existing theoretical framework to inform analysis which allowed us to go further than existing discourses on the anticipated acceptability of psychological interventions in low resource settings. The semi-structured nature of these data collection methods allowed participants to raise issues and topics that were still relevant but not encapsulated in the topic guides. Our study team included a wide range of expertise including lived, clinical and methodological experience from the UK and Indonesia and the structured approach to data analysis inherent to framework analysis supported the inclusion of these multiple viewpoints and ensured our analysis remained as grounded as possible in local contexts. Data was collected by local researchers and in local languages. Interviews/focus groups took part in accessible community venues which further increased accessibility.

Participants were recruited primarily through existing healthcare and community networks and may therefore differ from individuals who remain socially isolated, highly stigmatised, disconnected from services, or otherwise difficult to reach. Findings should therefore be interpreted as reflecting a broad range of stakeholder perspectives rather than population-level views. All data was collected in Java, Indonesia and the findings reported here may not reflect the views of those from other islands in the archipelago. Future research could explore the transferability of these findings to these groups. As this study was undertaken prior to intervention delivery, findings reflect anticipated rather than experienced acceptability. Stakeholder views may therefore change following direct exposure to intervention content, processes and implementation realities. Whilst requisite checks were made to ascertain the accuracy of transcripts in line with best practice guidance, some issues remained with translated transcripts and team members frequently had to revisit transcripts in the original language.

Future implementation studies should examine the transferability of these findings across Indonesia’s diverse provinces and assess how Kader training influences consistent delivery, sustainability and scale-up. Importantly, this study provides a foundational contribution to the evidence base on how low-intensity psychological interventions could be culturally adapted and integrated within community health systems in Indonesia, while demonstrating the value of applying knowledge mobilisation principles throughout the co-development process.

## Conclusion

This study underscores the complex interplay of knowledge gaps, cultural values, trust, stigma, and structural constraints in shaping the anticipated acceptability of low intensity psychological interventions in Java, Indonesia. This study also contributes novel insights into how cultural value alignment, role ambiguity beyond formal health workers, gendered perceptions of burden, and tension for change may interact to shape the anticipated acceptability of low-intensity psychological interventions. Addressing these challenges requires a contextually tailored, culturally congruent, and community-grounded approach that prioritises mental health literacy, builds trust, mitigates stigma, and supports Kader and health workers operationally. Such efforts are vital to improving mental health care access and outcomes in resource-limited settings

## Supporting information

S1 Appendix Comparing stakeholder perspectives across acceptability framework domains. (DOCX)
