# Correction: Stakeholder perspectives on the anticipated acceptability of low-intensity psychological interventions in Java, Indonesia: A theoretically framed qualitative study

**DOI:** 10.1371/journal.pone.0362386

**Published:** 2026-11-05

**Authors:** Herni Susanti, Novitri Nurimani Asha, Penny Bee, Heni Dwi Windarwati, Budi Anna Keliat, Atik Puji Rahayu, Amy Blakemore, Afni Handayani, Mochamad Ali Sodikin, Helen Brooks

In the Methods subsection of the Abstract, there is an error in the first sentence. The correct sentence is: Using a qualitative descriptive design guided by the Theoretical Framework of Acceptability (TFA), we conducted semi-structured interviews (n = 33) with people experiencing anxiety and depression and 21 focus groups with families (n = 34), health workers (n = 33), kader (lay health workers, n-22) and community/religious/traditional leaders (n = 18) across 12 sites in Java.

In the Results section of the Abstract, there is an error in the fifth sentence. The correct sentence is: Trust in lay health workers was high in rural areas but more variable in urban contexts highlighting that acceptability was contingent on interacting social, cultural and structural factors rather than inherent to the invention itself.

In the Introduction, the following sentence is missing after the second sentence of the second paragraph: Low-intensity psychological interventions (LIPIs) are structured, evidence-based approaches (e.g., behavioural activation and cognitive restructuring) delivered through guided self-help by non-specialists.

In the Introduction, the following sentence is missing after the third sentence of the second paragraph: Such approaches are structured and protocol-driven, in contrast to the more open-ended and non-directive nature of general counselling.

In the Introduction, there is an error in the second and third sentences of the fifth paragraph. The correct sentences are: Despite recent economic growth and attainment of middle-income status, long-standing deficits in treatment access persist (19) highlighting the limited translation of economic gains into investment in mental health systems. At 2.69–3.12% of the GDP in 2022–2023, Indonesia’s total health expenditure is one of the lowest of all LMICs, and the smallest among the Association of South-east Asian Nations (ASEAN) where the regional average is around 4% [20,21].

In the Design subsection of Materials and methods, there are errors in the first paragraph. The correct paragraph is: This multi-site study employed a qualitative descriptive design to provide a minimally interpretative, practice relevant account of stakeholder perspective. This was combined with the Theoretical Framework of Acceptability (TFA) which provided a structured lens with which to examine acceptability without the need for theory development or deeper interpretative analysis (24)Data was collected in 12 sites across four non-special region provinces, i.e., East, West and Central Java, and Banten in Java, Indonesia. Study sites were purposively selected to ensure diversity in geographical location, urban–rural context (defined by Indonesian National Population Census criteria), and health system capacity across Java, allowing for comparison of acceptability across varied implementation contexts.

In the Sample and recruitment subsection of the Materials and methods, there are errors in the first paragraph. The correct paragraph is: Participants were identified through purposive and network-based sampling via primary care services, community organisations, and professional contacts within study sites. As a multi-site qualitative study, a single sampling frame was not used, and response rates were not systematically recorded. Participants (aged 18 and over) were purposively sampled based on the criteria outlined below to ensure diversity in terms of geographical area, gender, age and diagnosis. Data collection commenced until data saturation was reached. This was defined as the point at which no new concepts relating to the research question and TFA constructs were identified and was agreed at ongoing team discussion in weekly meetings, assessed both within and across stakeholder groups, while remaining within the parameters of the sample size agreed with study funders (20–25 per stakeholder group).

In the Data Collection subsection of the Materials and methods, the following sentences are missing after the second sentence of the first paragraph: Topic guides were developed and informed by the TFA, with questions designed to explore key domains of acceptability while allowing flexibility for participants to raise context-specific issues. Guides were developed in collaboration with local partners, reviewed by our CEI advisory panel and refined iteratively during data collection to ensure cultural relevance and sensitivity. The guides were also designed to capture broader aspects of participant experience relevant to the wider programme of work, with additional components reported elsewhere.

In the Participants subsection of the Materials and methods, there are errors in the second sentence: The correct sentence is: These including 33 people with anxiety and depression, 34 family members, 33 health workers, 22 Kader, 5 Religious Healers, 11 Traditional Healers, 2 Community Leaders.

Tables 1 and 2 were uploaded incorrectly. Please see the correct Tables 1 and 2 here.

There are errors in the People with anxiety and depression subsection of the Materials and methods. The correct subsection is as follows:

## People with anxiety and depression

Most individuals with anxiety and depression were female and aged 18–75 years. While the majority had accessed some form of mental health care ranging from public health centres and hospitals to traditional or religious services, a small number had never sought care. Educational attainment was generally low, with around half having completed only primary or middle school.

### Family members

Family members, predominantly female and aged 26–69 years, included a range of caregiving roles such as parents, spouses, siblings, and friends. Caring experience ranged from 6 months to 5 years. Like patients, most caregivers had relatively low educational attainment and reported accessing diverse formal and informal mental health supports, often drawing on community and spiritual networks.

### Health workers

Health workers were mainly female, aged 25–59 years and represented a range of professional roles including general practitioners, nurses, midwives, pharmacists, and community health leaders. Most had over five years’ of professional experience working. Lay health workers (‘kader’) were also predominantly female, aged 30–59 years, and typically had several years of experience, though some were relatively new. Their educational backgrounds varied, but most had completed middle or high school. These profiles are consistent with prior evidence of kader roles in Indonesian mental health services.

### Community, religious and spiritual leaders

Community, religious, and spiritual leaders were mostly male, aged 23–60 years and commonly served as traditional healers or religious figures with longstanding community involvement. Their roles centred on spiritual guidance, healing practices, and community support.

In the Lack of adequate mental health literacy as a threat to anticipated intervention acceptability subsection of the Results, there are errors in the second paragraph. The correct paragraph is: This limited awareness contributed to low intervention coherence across stakeholder groups in relation to the intervention’s content, aims and underlying mechanisms. Whilst researchers provided an overview of the intervention, participants commonly conflated LIPIs with general counselling services rather than aligning them with principles of guided self-help. The appeared to reflect not only familiarity with school- or health service-based counselling which are the most accessible and recognisable forms of mental health care in Indonesia but also broader challenges for participants in distinguishing between structured, skills-based self-help interventions and more relational, directive and intensive models of care. This conflation has potential implications beyond just intervention coherence suggesting that if not addressed LIPIs may be interpreted and delivered in ways that diverge from intended mechanisms.

In the Strength of perceived need and alignment with individual and cultural values as drivers for anticipated acceptability subsection of the Results, the following sentences should be added after the third sentence of the second paragraph. The sentences are: This appeared to reflect a more nuanced interplay between individual and social expectations. In the Indonesian context, where help-seeking is heavily shaped by stigma, family relationships and concerns about burdening others, self-directed approaches may offer an acceptable alternative to formal support. However, this emphasis on autonomy in participant narratives occurred alongside the enduring value of family and community support, suggesting that the acceptability of LIPIs may depend on their ability to complement rather than replace existing social structures. This indicates that autonomy is not understood as purely individual but is relationally negotiated within social and cultural contexts.

In the Strength of perceived need and alignment with individual and cultural values as drivers for anticipated acceptability subsection of the Results, the following sentences should be added after the third sentence of the fifth paragraph. The sentences are: This may suggest that there is potential for interventions to be understood through different explanatory frameworks. While LIPIs are predicated on structured psychological principles, religious and traditional conceptualisations often emphasise spiritual, moral or social causes meaning participants may interpret the intervention in ways that align with these beliefs. Whilst this might support engagement, it may also lead to differences in how this intervention is delivered or understood in practice.

In the Stigma, privacy and the social risks of engaging with low-intensity psychological interventions, there is an error in the first sentence of the first paragraph. The correct sentence is: Pervasive individual-, family-, and community-level stigma directed towards people experiencing mental health difficulties emerged across stakeholder groups, reflecting broader social and cultural norms and raising important considerations in relation to ethicality.

In the Discussion, the following sentences should be added after the first sentence of the first paragraph. The sentences are: Our findings also provide insight into how TFA constructs operate within LMIC settings, highlighting the need for contextual interpretation to capture culturally embedded understandings of acceptability. Importantly, acceptability was not a fixed property of the intervention, but emerged as conditional and socially negotiated, shaped by interactions between trust, privacy norms, gendered burden, and the perceived legitimacy of community-based delivery.

In the Discussion, the following sentences should be added after the fourth sentence of the first paragraph. The sentences are: This study focuses specifically on acceptability as one dimension of implementation. Broader issues of feasibility and sustainability are being examined in parallel work within the wider programme and will be reported separately.

In the Discussion, there are errors in the second paragraph. The correct second paragraph is: Low awareness of mental health diagnoses, particularly anxiety and depression, and of non-pharmacological interventions was evident across transcripts, reflecting findings from other low-resource settings (32). This limited mental health literacy, combined with the novelty of the proposed intervention, contributed to low intervention coherence across stakeholder groups (24). In particular, guided self-help was frequently conflated with counselling. This conflation has important implications for the assumed mechanisms of action, as participants appeared to expect more directive, relational, and intensive input than is characteristic of low-intensity, skills-based approaches. Such differences point to potential challenges for intervention fidelity, including deviations in delivery style, intensity, and the balance of responsibility between facilitator and participant, which may arise in future implementation. These misalignments may also influence engagement and outcomes if the intervention is experienced as insufficiently supportive or misaligned with expectations (32). These findings further highlight how existing service models and culturally embedded expectations of care shape how new interventions are understood. In contexts where counselling represents the most familiar form of psychological support, novel low-intensity approaches may be interpreted through this lens. This underscores the need for clearer communication around intervention aims and underlying mechanisms, alongside contextual adaptation and proactive strategies to support fidelity such as targeted training, supervision, and monitoring when introducing low-intensity approaches in LMIC settings. Similar misalignments have been identified in high-income settings as barriers to engagement (33), suggesting that tailored preparation of stakeholders is critical to aligning expectations prior to implementation.

In the Discussion, the following sentences should be added after the third sentence of the fourth paragraph. The sentences are: In the Indonesian context specifically, these values are embedded within religious teachings and communal norms that emphasise personal responsibility, mutual support (gotong royong) and maintaining social harmony. As a result, guided self-help interventions may be viewed positively because they enable people to manage distress without the need to disrupt or burden family or broader communities. Gender norms may also play a role with more discreet home-based forms of support potentially seen as more appropriate for women or those with restricted mobility of social roles.

In the Discussion, there are errors in the fifth paragraph. The correct fifth paragraph is: The cultural context in Java, Indonesia, significantly shaped perceptions of acceptability. Existing trust in Kader was a key facilitator, particularly in rural areas where Kader were seen as embedded, familiar, and accessible members of the community. In contrast, participants from more urban areas expressed greater concerns about trust, particularly regarding Kader’s ability to maintain confidentiality and demonstrate professionalism. These differences highlight how expectations of care vary across contexts, with urban participants placing greater emphasis on formalised and discreet models of support. Importantly, our findings further suggest that the modality of intervention delivery not just its content may conflict with norms of discretion, even in contexts where trust in providers is high. For example, repeated home visits from Kader may increase visibility to neighbours, potentially signalling engagement with mental health services and heightening concerns about gossip and social judgement. Concerns about social judgement have been widely identified as barriers to help-seeking (39), and were reflected here as part of broader community-level stigma relating to mental health, which participants perceived as detrimental to engagement and uptake (40).Together, these findings highlight that both trust and discretion are contextually shaped and critical to the acceptability of Kader-led delivery. Differences between rural and urban settings indicate that Kader-based models may require adaptation to meet varying expectations around confidentiality, professionalism, and visibility particularly in more urbanised areas. This has important implications for future implementation, suggesting a need for context-sensitive delivery approaches that minimise unintended disclosure while maintaining accessibility. Delivering tailored, community-based awareness-raising initiatives, potentially in partnership with trusted community figures such as religious and spiritual leaders, may also help to reduce stigma and enhance acceptability (41).

In the Discussion, a sentence should be added after the second sentence of the sixth paragraph. The sentence is: These concerns were closely linked to the voluntary or under-resourced nature of Kader roles and the broader pressures on primary care staff, raising questions about the sustainability and scalability of delivery over time.

In the Discussion, a sentence should be added after the fifth sentence of the sixth paragraph. The sentence is: This suggests that participation was not solely an individual decision, but was mediated through household decision-making processes, reflecting gendered constraints on autonomy and access to support.

In the Strengths and limitations subsection of Discussion, there is an error in the first sentence of the second paragraph. The correct sentence is: Participants were recruited primarily through existing healthcare via clinical judgement/primary care records rather than standardised diagnostic or screening measures and community networks and may therefore differ from individuals who remain socially isolated, highly stigmatised, disconnected from services, or otherwise difficult to reach.

In the Strengths and limitations subsection of Discussion, the following sentences should be added after the seventh sentence of the second paragraph. The sentences are: This process of cross-checking ensured that any ambiguities were resolved and did not materially influence the interpretation of key themes. It is also worth noting that we did not include representatives from district or provincial health offices. Such perspectives on system-level planning, feasibility, and sustainability will be important areas for future research and will be a focus of later stages of the wider programme of work.

**Table 1:** Breakdown of participants across sites and groups.

Province	Site	People with anxiety and depression	Family members	Kader	Religious Leader	Traditional Healer	Community Leader	Health Professionals	Total
Banten	**Tangerang city**								
	Cipadu	5	5	3	0	0	2	5	20
West Java	**Bogor District**								
	Gn Bunder 1	3	3	3	0	0	0	4	13
	Gn.Bunder 2	4	3	0	4	0	0	3	14
	Cibunian	3	3	3	0	0	0	3	12
Central Java	**Magelang District**								
	Borobudur	2	3	0	0	2	0	3	10
	Mungkid	2	2	0	0	3	0	2	9
	**Semarang City**								
	Bandarharjo	2	3	2	0	2	0	5	14
	Bulu lor	3	2	1	0	0	0	0	6
East Java	**Jombang District**								
	Kabuh	3	2	1	0	2	0	1	9
	Dukuh Klopo	2	3	4	0	2	0	2	13
	**Malang District**								
	Bantur	0	3	3	0	0	0	3	9
	Bululawang	4	2	2	1	0	0	2	11
	**Total**	**33**	**34**	**22**	**5**	**11**	**2**	**33**	**140**

**Table 2:** Participant demographics.

Stakeholder group	n	Male, % (n)	Female, % (n)	Mean age (years)	Age range (years)
**People with anxiety and depression**	33	18.2 (6)	81.8 (27)	37.42	18–75
**Family members**	34	32.4 (11)	67.6 (23)	46.88	26–69
**Health workers**	33	24.2 (8)	75.8 (25)	39.85	25–59
**Kader**	22	18.2 (4)	81.8 (18)	45.68	30–59
**Community, traditional and religious leaders**	18	77.8 (14)	22.2 (4)	46.4	23–60
