# Supplementary material for: Stakeholder perspectives on the anticipated acceptability of low-intensity psychological interventions in Java, Indonesia: A theoretically framed qualitative study
Source: PLoS One. 2026 Sep 29;21(9):e0358869. doi: 10.1371/journal.pone.0358869 (PMC13623157; doi:10.1371/journal.pone.0358869)
Supplement: Supplementary file 1 [file pone.0358869.s001.docx]

| **TFA Component** | **Health Workers (Nurses, GP, Pharmacist**) | **Family Members** | **Kader, Spiritual & Religious Leaders** | **People with Anxiety / Depression** | **Key Differences / Notes** |
| --- | --- | --- | --- | --- | --- |
| **Affective Attitude** | Participants generally acknowledged the clear need for the intervention and expressed strong motivation to contribute to or participate in it. At the same time, they voiced concerns that the program might place additional burdens on already overstretched cadres or families. These concerns underscored the importance of implementing the intervention in a way that minimizes extra workload and resource demands, ensuring sustainability and continued engagement. | Families expressed positive views toward LIPI, describing it as a needed, useful, and innovative intervention. They emphasized its benefits for both mental and physical well‑being, noting that it not only makes service users happier but also provides meaningful support through listening and accompaniment. | Participants generally viewed the intervention positively, noting that it was consistent with religious teachings and communal norms. This alignment reinforced trust and acceptance within the community, while also fostering a sense of collective responsibility. Religious and community leaders emphasized that the program complemented existing values of compassion, solidarity, and mutual support, which helped reduce resistance and encouraged broader participation. | Participants expressed hope and optimism about the potential benefits of the program, noting its relevance and usefulness for community health. However, these positive expectations were tempered by concerns about stigma and fear of social judgment. Such worries reduced enthusiasm and limited the extent to which individuals felt comfortable engaging openly with the intervention. This tension highlights the importance of addressing stigma and ensuring that participation is framed in ways that protect dignity and foster acceptance. | Perceptions of the intervention varied across groups. Urban participants were more likely to express skepticism about Kader competence, particularly if they anticipated that sessions would be generic or lacking practical solutions. In contrast, families emphasized their motivation to engage, highlighting the potential of the program to provide much‑needed relief in caregiving responsibilities. Spiritual and religious leaders tended to be supportive, especially when the intervention was framed as consistent with faith‑based values and communal traditions. These mixed perspectives underscore the importance of tailoring implementation strategies to different stakeholder concerns and expectations. |
| **Burden** | Participants highlighted several challenges that could negatively affect the intervention’s implementation. Staff were described as overworked, with few professionals available and minimal incentives for cadres, raising concerns about sustainability. Stigma remained a significant barrier, with patients withdrawing, families keeping conditions secret, and health workers being labeled as “mental health nurses,” which discouraged engagement. Geographical barriers, such as remote areas and poor road conditions, further limited access. Confidentiality concerns also emerged, with cadres sometimes perceived as “blabbermouths,” undermining trust. Finally, depression was often misunderstood as a sign of weak mentality, underscoring the need for additional education and awareness‑raising to improve acceptance and reduce misconceptions. | Families acknowledged the important role of cadres but emphasized that the time and effort required of them is substantial, particularly when serving communities spread across large geographical areas. This perception raised concerns about the sustainability of Kader involvement, as extensive travel and workload demands could limit their capacity to provide consistent support | Community leaders emphasized that significant cognitive and emotional effort may be required to ensure the intervention’s success. They noted the challenge of convincing depressed patients to engage, building awareness among families and support systems, and overcoming stigma and resistance within the community. Leaders also highlighted the need to train cadres, who often begin with low baseline knowledge and are sometimes poorly selected, to strengthen their competence. Finally, they stressed the importance of informing service users that they need help, underscoring the role of education and communication in fostering acceptance and participation. | Participants identified significant barriers to participation, noting that the intervention required considerable time and travel, which was difficult to balance with household responsibilities. Women and low‑income participants were particularly affected, as they often needed explicit permission from family members to engage. These constraints reduced accessibility and highlighted the importance of designing implementation strategies that account for gendered expectations, economic limitations, and family dynamics to ensure equitable participation. | Participants highlighted several factors influencing perceptions of burden. Existing Puskesmas networks were seen as potentially reducing workload if effectively activated. Some families found home visits and flexible timing arrangements non‑burdensome, while others described talking as demanding compared to enjoyable activities, which were perceived as low‑effort. Family self‑learning, such as searching for information online, was noted to add indirect burdens on program deliverers, who must anticipate and correct misinformation. In many transcripts, certain issues were not discussed or explicitly identified, suggesting variability in how burdens were perceived and articulated across groups. |
| **Ethicality** | This group emphasize individual and collective ethical alignment: 1:1 face-to-face delivery is preferred to avoid group exposure; cadres are well-placed due to closeness, trust, and patient knowledge but require training; confidentiality is critical to prevent gossip and judgment; stigma blocks access to Puskesmas, psychiatry, and home visits; economic aid and socialization are morally expected; religious leaders need empathy training. | Families expressed strongly positive views of LIPI, emphasizing its ethical alignment at both individual and collective levels. They highlighted that delivery must be carried out by trusted, respected, and mentally strong individuals, including cadres, health workers, family members, or religious leaders. Home visits and voluntary participation were considered essential, with family support viewed as mandatory for success. A subtle, non‑forceful approach was preferred, alongside formats that respected cultural sensitivities—such as same‑sex or age‑specific groups, or individual sessions. Families also highlighted the need for professional supervision of cadres to ensure quality and accountability. Religious content and community closeness were seen as reinforcing values, further strengthening trust and acceptance of the intervention. | Community leaders expressed positive views of LIPI, emphasizing its ethical alignment at both individual and collective levels. They noted that the intervention resonates with cultural and religious values, including story‑sharing, Islamic health efforts, and prayer before acts. Leaders highlighted the importance of home or near‑community access, delivered by trusted local figures such as cadres, community leaders, and religious representatives. Privacy boundaries were considered essential to protect dignity, while family and community inclusion were seen as critical for sustainability. Incentives for cadres, clarity that services are free of cost, and voluntary participation were all expected to ensure fairness and trust. These elements reinforced perceptions of LIPI as culturally appropriate and ethically grounded. | Service users expressed mixed views of LIPI. On one hand, they found it ethically aligned with self‑care values and cultural practices of talking, autonomy, empathy, and privacy. They considered it particularly suitable for individuals facing mild mental health issues, limited social support, or economic stress. Preferences included an individual format, free access, health worker leadership, and weekly sessions with flexibility in choosing the place of delivery. On the other hand, some participants noted conflicts with prevailing social norms and concerns about family honour, alongside fears of being labeled “mentally ill.” These tensions highlight both the promise of LIPI in meeting user needs and the barriers posed by stigma and cultural expectations that must be addressed for broader acceptance. | Participants raised several negative concerns about LIPI’s delivery and suitability. Cadres were sometimes perceived as unfit to address depression due to inferiority dynamics and low trust compared to health workers. Financial needs often took priority over participation, while middle and upper classes tended to avoid Puskesmas altogether. Stigma remained a major barrier, blocking access and making the program unsuitable for severe or elderly cases. Traditional or religious approaches were often preferred before medical interventions. Cadres were frequently seen as unqualified, though some valued their role as lay insiders. Preferences varied between group and individual formats, with stigma disproportionately affecting older or unemployed participants. Contrasting views highlighted delivery conflicts: whether family inclusion was essential or problematic (due to isolation or shackling), whether direct home information reduced embarrassment or increased it, whether booklets were ineffective compared to direct recommendations, and whether supernatural beliefs conflicted with the medical model. These tensions underscored the complexity of implementation and the need for careful adaptation to diverse community expectations. |
| **Intervention Coherence** | This group demonstrated limited awareness of LIPI, with many confusing it with general mental disorders rather than recognizing its distinct model. Their understanding of symptoms and the specific framework underpinning LIPI was often incomplete, which reduced confidence in implementation and hindered accurate communication with patients and families. This gap highlights the need for targeted training and orientation to ensure health workers can differentiate LIPI from broader mental health concepts and apply it effectively in practice. | Families demonstrated low awareness of LIPI, frequently confusing it with counseling, support groups, or recreational activities. They tended to rely heavily on guidance from health workers and cadres to understand the program. While families recognized that LIPI should involve talk combined with treatment rather than medication alone, their limited knowledge highlighted the need for clearer communication and orientation. Health workers—particularly midwives—were viewed as ideal deliverers due to their credibility, familiarity, and established relationships with families. These perceptions suggest both gaps in awareness and opportunities to strengthen trust and uptake through professional leadership and targeted education | Community leaders demonstrated low to moderate awareness and coherence regarding LIPI. They were often unclear on its intervention mechanisms and sometimes conflated it with spiritual practices. Many perceived LIPI as community‑based, counseling, or clinic‑oriented rather than as a self‑help or home‑visit model. Leaders tended to misinterpret it as professional‑led, while simultaneously seeing no clear role for themselves beyond non‑medical contributions. Their understanding of program content was blurry, though they did grasp aspects related to activity direction and lifestyle or cognitive components. These gaps highlight the need for clearer communication and training to ensure leaders can accurately position LIPI within community health structures and recognize their potential role in supporting implementation. | Service users demonstrated low to moderate awareness of LIPI, reflecting limited mental health literacy. Mild distress was often not recognized as requiring intervention, and the program was frequently conflated with general counseling. Despite these gaps, users understood that talking could lead to calm and relief, though they emphasized that practice beyond reading was necessary for effectiveness. They perceived LIPI as targeting individuals who were anxious or sad, and highlighted the importance of clear language and local delivery as logical features that would make the intervention accessible and acceptable. These findings underscore both the potential resonance of LIPI with user expectations and the need for improved education to distinguish it from broader counseling practices | At the same time, certain participants saw no barriers to implementation, recognizing that LIPI fit well with mental health needs and could enable collaboration with specialists for severe cases. These mixed perspectives highlight both the strengths of professional involvement and the challenges of ensuring broader community understanding and trust. |
| **Opportunity Costs** | Health workers expressed moderate views of LIPI’s implementation, noting that while supervision and training are necessary, they also add to workload pressures. Resource and access barriers were highlighted, including reliance on personal vehicles, poor road conditions, and the absence of public transportation options. Time and financial costs associated with building awareness and reducing stigma were seen as significant challenges. Additionally, booklets used in the program require cadres to achieve mastery, which further increases demands on training and oversight. These factors illustrate the balance between the program’s potential benefits and the practical burdens faced by health workers in ensuring effective delivery. | Families expressed moderate views of LIPI’s delivery. While time demands and household disruption were noted as challenges, Posyandu delivery was strongly preferred because it saved time, travel, and money compared to distant Puskesmas or hospitals. Families emphasized their willingness to invest effort and resources to support patients’ mental peace, including practices such as buying what the patient wants (nyerateni). These perspectives highlight both the practical barriers families face and the cultural commitment to caregiving, suggesting that community‑based delivery through Posyandu may enhance accessibility and acceptance. | Community leaders expressed moderate views of LIPI’s implementation. On one hand, they noted that time demands were manageable and cultural expectations minimal, suggesting feasibility in principle. On the other hand, they emphasized the high resource demands required for effective delivery, including funding to support multi‑stakeholder involvement. Deliverers were expected to invest significant time and emotional effort, particularly in deep listening and addressing personal issues raised by participants. These factors highlight both the relative ease of aligning with cultural norms and the substantial burden of resources and emotional labor required for sustainable implementation. | They noted burdens such as lost work, household disruption, and cultural expectations when hosting sessions. While they strongly expected the program to be free or low‑cost, they also faced practical challenges including travel and childcare responsibilities if sessions were distant, as well as the need for materials such as pamphlets and rooms. Time away from household or occupational duties was seen as a significant disruption. Poor delivery was perceived as potentially leading to emotional costs, were identified as barriers to consistent access and quality. These perspectives highlight both the feasibility of LIPI under certain conditions and the risks of added burdens if delivery is not carefully managed. | Perceptions of LIPI’s accessibility were mixed. On the positive side, participants noted that it reduced prior barriers such as insurance requirements, and when delivered locally or on a monthly basis, burdens were minimal. Practical help was seen as offering high value. However, when services were distant, travel created significant time and financial burdens, limiting feasibility. Many transcripts did not discuss these issues in detail, and certain aspects were not identified at all, suggesting variability in awareness and articulation across groups. These findings highlight both the potential of LIPI to lower systemic barriers and the persistent challenges of distance and cost that require careful planning. |
| **Perceived Effectiveness** | Health workers expressed positive views of LIPI, perceiving it as an intervention that fills existing service gaps. They emphasized the role of cadres as an “extension of hands,” supporting delivery and outreach. One‑to‑one, face‑to‑face sessions were considered the most effective format, as they allow open expression and build trust. Health workers highlighted LIPI’s potential to reduce anxiety and depression, prevent emerging issues, and increase awareness within communities. Campaigns were seen as essential to promote understanding and uptake, ensuring that the intervention reaches those who need it most. | Families expressed positive hopes that LIPI would reduce the caregiving burden they often face. The intervention was perceived as effective for addressing anxiety and depression across both age groups and urban/rural settings. Families noted that participation lightened their responsibilities, helped individuals release “stuffy” thoughts, and improved behaviour. Success was seen as highly dependent on the qualities of people who deliver : gentle, patient, non‑emotional, trusted, and respected figures with strong communication skills were considered essential for building confidence and ensuring meaningful outcomes. These perceptions underscore LIPI’s potential to ease family strain while promoting mental health improvements across diverse contexts. | Community members expressed moderate views of LIPI’s effectiveness, noting its potential if aligned with cultural and religious principles. The intervention was perceived as positive and widely applicable, particularly when delivered in a one‑to‑one personal format that created a strong “connection of two hearts.” Listening and sincerity were emphasized as essential qualities of deliverers. Participants reported that the approach helped calm and comfort service users, supported long‑term illness management such as diabetes, and improved mental health through effective communication. These perspectives highlight both the promise of LIPI and the importance of cultural sensitivity and interpersonal trust in ensuring its success. | Service users expressed, combining hope for improvement with some doubts about Kader competence, particularly in urban settings. They emphasized its practical relevance for mild symptoms and generally perceived it positively. Listening and sharing were seen as effective strategies that created a sense of calm and relief, complementing personal efforts toward recovery. Gentle communication and the delivery of homework tasks were preferred approaches, while tools and booklets were valued for supporting understanding and reinforcing practice. These perspectives highlight both optimism about LIPI’s potential and the need to strengthen Kader training and delivery quality to ensure consistent effectiveness | Effectiveness was seen as limited for older adults or those facing economic problems, while booklets were often ineffective for individuals with low literacy or rejected when perceived as irrelevant. Awareness gaps blocked uptake in many cases. Some participants doubted Kader‑only delivery, viewing the intervention as less suitable for older adults or introverted individuals, and some prioritized medication instead. Effectiveness was perceived to depend on autonomy, tailored support, and respect for privacy, with acceptability varying by personal circumstances. The intervention was considered less effective for individuals unable to be open or honest. Government and policy support were viewed as essential for success, though many transcripts did not discuss this aspect in detail. Booklets were seen as mixed: helpful for mild depression and younger people, but less suitable for severe cases. Importantly, no groups were considered excluded, though discussion on this point was limited. |
| **Self-Efficacy** | Perceptions of LIPI’s effectiveness and implementation were mixed, shaped by clinical, cultural, and structural factors. Health professionals noted that anxiety posed particular challenges due to its physical symptoms and patients’ reluctance to accept psychological explanations. In contrast, depression was seen as more treatable through psychosocial approaches. Confidence in delivering LIPI was closely tied to the availability of standardized psychoeducational materials—many professionals expressed hesitation to print or share information without clear templates, fearing misinformation.  Training from academic experts was highly valued, especially in preparing cadres. However, cultural and religious beliefs within the wider community were seen as major barriers to understanding mental illness, with many attributing symptoms to non-medical causes. This misunderstanding extended to depression severity, where participants struggled to distinguish between mild and severe cases, often normalizing symptoms due to lack of awareness.  Participants emphasized that increased knowledge should be collective—shared not only among health workers but also with community leaders, religious healers, and the general public. Guidebooks and manuals were seen as essential tools to boost self-efficacy and ensure consistent, confident delivery of LIPI across diverse settings. | Perceptions of LIPI’s viability were moderate, with participation seen as possible only if families permitted. Both collective and individual understanding of mental health was low, as families and patients had limited knowledge of causes, trajectories, and treatments. This lack of awareness meant LIPI was often not viewed as a viable option. Participants struggled to differentiate between common and severe illness, reinforcing the belief that specially trained professionals such as psychologists were needed rather than lay or Kader support. These findings highlight the importance of strengthening mental health literacy and clarifying the role of cadres to build trust and acceptance of LIPI. | Cadres and community members expressed moderate views of LIPI’s implementation. Engagement was seen as possible when cadres were respected and their roles clearly defined. However, both individual and collective efficacy were perceived as low, with embarrassment acting as a barrier to participation. Uncertainty about effectiveness, regional variation in delivery, and the absence of standardized operating procedures contributed to fear and poor management. Younger service users were particularly difficult to reach due to the age gap with cadres, highlighting generational challenges in communication and trust. Sustainability of the intervention was also raised as a concern, underscoring the need for stronger systems, clear guidance, and long‑term support. | Participants expressed moderate views of LIPI’s potential. Engagement was constrained by family permission, time demands, and prevailing social norms, though collective support was seen as a way to strengthen participation. Confidence in the intervention was conditional on the presence of a trusted health worker, the availability of a safe space, and clear practical relevance. When these conditions were met, participants were open to trying the intervention. Reflections were primarily individual‑focused, highlighting personal experiences and needs rather than collective perspectives. These findings suggest that trust, safety, and contextual relevance are critical to building confidence and sustaining engagement. | self‑efficacy was low when privacy or trust issues, shyness, or stigma were present, and higher among individuals who were self‑reliant or resistant to stigma. Support from religious leaders (kyai) was helpful for some but not universally effective. A few participants expressed strong confidence, noting that ‘no barrier, everyone can do it’ when adequate guidance was provided. Importantly, this theme was not widely discussed across transcripts, suggesting uneven awareness or articulation. |
