# Supplementary material for: Stakeholder perspectives on the anticipated acceptability of low-intensity psychological interventions in Java, Indonesia: A theoretically framed qualitative study
Source: PLoS One. 2026 Sep 29;21(9):e0358869. doi: 10.1371/journal.pone.0358869 (PMC13623157; doi:10.1371/journal.pone.0358869)
Supplement: Supplementary file 2 [file pone.0358869.s002.docx]

**Manuscript title:**
*Stakeholder perspectives on the acceptability of low-intensity psychological interventions in Indonesia: A theoretically framed qualitative study*

**General comments**

This manuscript addresses an important and timely topic on low-intensity psychological interventions (LIPIs). However, there are several major methodological and conceptual issues that need to be addressed before the manuscript can be considered for publication.

**Major comments**

1. The scope of this paper focuses solely on *acceptability*. What was/were about the *feasibility* and *sustainability*?
2. The term *low-intensity psychological interventions* should be clearly defined earlier in the Introduction (around line 68). A concise operational definition, including examples relevant to the Indonesian context, would improve readability and conceptual clarity. Also, the manuscript does not sufficiently discuss what kind of LIPI and how it would be delivered. In the Methods section, author should add amore details on the LIPI: who would deliver the intervention (and eligibility criteria), the dosage and intensity, that was intended by authors and asked to the participants. These are central to assessing acceptability and feasibility.
3. The inclusion criteria (Methods, line 164) are insufficiently specified. Criteria should be clearly defined **for each stakeholder group**, including:
   - Health workers (e.g., nurses, psychologists, psychiatrists, general practitioners)
   - Patients (e.g., setting: hospital vs. primary care; diagnostic status)
   - Family members (e.g., parents, spouses, caregivers)
   - Community leaders (clear definition and role)
   - Traditional healers (clear definition and scope of practice)

The use of “OR” in the inclusion criteria is inappropriate and should be revised.

4. The rationale for including traditional healers is unclear. Are they potential deliverers of LIPI, referral agents, or contextual stakeholders? This should be clearly explained.
5. It is also unclear why representatives from district or provincial health offices (policy makers or programme planners) were not included, given their central role in implementation and sustainability. The authors should justify this omission or discuss it as a limitation.
6. The exclusion criteria are not clearly described. It should be specified **for which stakeholder groups** these criteria applied.
7. The manuscript didn’t state if purposive sampling was used, and the specific criteria guiding purposive selection are not clearly explained. Please clarify how participants were selected and what characteristics were prioritized.
8. The description of recruitment raises several questions:
   - What registry or sampling frame was used to identify potential participants?
   - What was the response rate?
   - Were there refusals, and if so, what were the reasons?
   - How did the authors ensure that participants were appropriate representatives of their stakeholder groups?
9. The criteria for deciding whether participants joined in-depth interviews or focus group discussions are unclear. Please clarify how and why these different data collection methods were used.
10. **Table 1 (Study locations).** Several city names are listed, but the rationale for selecting these cities is not explained. Please justify the choice of study sites.
11. **Table 2 (Participant characteristics).** Table 2 is confusing and would benefit from reorganisation. General characteristics (e.g., age, gender) should be separated from stakeholder-specific variables. This table, along with the accompanying text, should be moved from the Methods section to the Results section and the text should be substantially shortened.
12. The study refers to seven components (*affective attitude, burden, ethicality, intervention coherence, opportunity costs, perceived effectiveness, and self-efficacy*) as the analytic framework. However, the Results section is not structured according to these components. The authors should explain this deviation or revise the Results to align clearly with the stated framework.
13. The manuscript does not clearly describe the interview or FGD guides used. Please specify:
    - What instruments were used
    - Whether they were adapted from existing tools
    - How their validity or credibility was ensured
14. **Lines 315–316:** The statement that “most participants, particularly non-professionals, had limited awareness of mental health” appears misleading. Patients diagnosed with depression or anxiety would be expected to have some awareness of these conditions. The quoted data seem to reflect *health workers’ perceptions* of patients’ awareness rather than patients’ own views. This interpretation should be revised.
15. LIPI is a complex concept and likely unfamiliar to lay participants. The Methods section should clearly describe **how LIPI was introduced during interviews**, how understanding was checked, and how misunderstandings were addressed.
16. The manuscript implies that depression and anxiety lead to stigma. Conceptually, stigma is more appropriately framed as a *determinant* of mental health conditions. This relationship needs clarification.
17. The quote stating that people with anxiety “don’t want anyone to know” and “try to look happy” appears to reflect misunderstanding of anxiety disorders. The authors should critically reassess whether this quote validly represents anxiety or reflects lay misinterpretation.
18. The quote describing people as “angry or emotionally unstable” lacks clarity. It is unclear whether respondents were referring to depression, anxiety, or other conditions. The authors should clarify this in the analysis and check the validity.
19. Although the study claims to explore acceptability of LIPI, many quotes and analyses do not clearly refer to LIPI itself. For example, statements such as “I think it is really very much needed” are vague, and it is unclear what “it” refers to. The analysis should more explicitly focus on LIPI.

**Minor comments**

1. Please conduct a thorough **grammar and language check**, particularly ensuring consistent use of the past tense in the Methods section.
2. Abstract (line 37): Please spell out *LIPI* at first mention.
3. Check punctuation and consistency throughout the manuscript.
4. The discussion of economic growth in the Introduction (lines 91–94) seems tangential. The Indonesian context should be introduced earlier and more directly linked to mental health system challenges.
