# Supplementary material for: Stakeholder perspectives on the anticipated acceptability of low-intensity psychological interventions in Java, Indonesia: A theoretically framed qualitative study
Source: PLoS One. 2026 Sep 29;21(9):e0358869. doi: 10.1371/journal.pone.0358869 (PMC13623157; doi:10.1371/journal.pone.0358869)
Supplement: Supplementary file 3 [file pone.0358869.s004.docx]

Dear Editor

**RE: PONE-D-25-65667: Stakeholder perspectives on the acceptability of low-intensity psychological interventions in Indonesia: A theoretically framed qualitative study**

We thank the editor and the reviewers for your careful and constructive feedback on our manuscript. We have revised the manuscript in response to all comments and believe these revisions have strengthened the clarity, rigour, and contribution of the paper. Below, we provide a point-by-point response to each comment and indicate where changes have been made in the revised manuscript**.**

**Editorial comments:**

**When submitting your revision, we need you to address these additional requirements.**

**1.Please ensure that your manuscript meets PLOS ONE's style requirements, including those for file naming. The PLOS ONE style templates can be found at**

[**https://journals.plos.org/plosone/s/file?id=wjVg/PLOSOne_formatting_sample_main_body.pdf [journals.plos.org]**](https://urldefense.com/v3/__https:/journals.plos.org/plosone/s/file?id=wjVg*PLOSOne_formatting_sample_main_body.pdf__;Lw!!PDiH4ENfjr2_Jw!GFqrB7o03x83g4bPbnNxGR7EdkciuYQkUswlPRyUblGIlgjuJ-4192X2h763iNcnsoCePBxSwcocpFfry3d9jI0wHQ$) **and**

[**https://journals.plos.org/plosone/s/file?id=ba62/PLOSOne_formatting_sample_title_authors_affiliations.pdf [track.editorialmanager.com]**](https://urldefense.com/v3/__https:/track.editorialmanager.com/CL0/https:*2F*2Fjournals.plos.org*2Fplosone*2Fs*2Ffile*3Fid=ba62*2FPLOSOne_formatting_sample_title_authors_affiliations.pdf/1/010f019e73d616b8-a4831803-5e6f-4d15-bfcc-2602e45d5a8a-000000/yON021VZczyQCjRs7XwA_P3TvAGhjPtp_JJgqYuFFok=258__;JSUlJSUlJQ!!PDiH4ENfjr2_Jw!GFqrB7o03x83g4bPbnNxGR7EdkciuYQkUswlPRyUblGIlgjuJ-4192X2h763iNcnsoCePBxSwcocpFfry3czvWIyQA$)

**2. Please include a complete copy of PLOS’ questionnaire on inclusivity in global research in your revised manuscript. Our policy for research in this area aims to improve transparency in the reporting of research performed outside of researchers’ own country or community. The policy applies to researchers who have travelled to a different country to conduct research, research with Indigenous populations or their lands, and research on cultural artefacts. The questionnaire can also be requested at the journal’s discretion for any other submissions, even if these conditions are not met.  Please find more information on the policy and a link to download a blank copy of the questionnaire here:** [**https://journals.plos.org/plosone/s/best-practices-in-research-reporting [journals.plos.org]**](https://urldefense.com/v3/__https:/journals.plos.org/plosone/s/best-practices-in-research-reporting__;!!PDiH4ENfjr2_Jw!GFqrB7o03x83g4bPbnNxGR7EdkciuYQkUswlPRyUblGIlgjuJ-4192X2h763iNcnsoCePBxSwcocpFfry3f1ADGdow$)**. Please upload a completed version of your questionnaire as Supporting Information when you resubmit your manuscript.**

Attached to resubmission as supporting information.

**3. Please note that funding information should not appear in any section or other areas of your manuscript. We will only publish funding information present in the Funding Statement section of the online submission form. Please remove any funding-related text from the manuscript.**

We have removed funding statement from the manuscript.

**4. Thank you for stating the following financial disclosure:**

**“This research was funded by the NIHR [Global Health Research for Sustainable Care for anxiety and depression in Indonesia (Award ID NIHR134638)] using UK international development funding from the UK Government to support global health research. The views expressed in this publication are those of the author(s) and not necessarily those of the NIHR or the UK government.”**

**Please state what role the funders took in the study.  If the funders had no role, please state: "The funders had no role in study design, data collection and analysis, decision to publish, or preparation of the manuscript."**

**If this statement is not correct you must amend it as needed.**

**Please include this amended Role of Funder statement in your cover letter; we will change the online submission form on your behalf.**

The statement is correct: The funders had no role in study design, data collection and analysis, decision to publish, or preparation of the manuscript.

**5. Please expand the acronym “NIHR” (as indicated in your financial disclosure) so that it states the name of your funders in full.**

**This information should be included in your cover letter; we will change the online submission form on your behalf.**

**National Institute for Health and Care Research (NIHR)**

**6. We note that you have indicated that there are restrictions to data sharing for this study. PLOS only allows data to be available upon request if there are legal or ethical restrictions on sharing data publicly. For more information on unacceptable data access restrictions, please see** [**http://journals.plos.org/plosone/s/data-availability#loc-unacceptable-data-access-restrictions [journals.plos.org]**](https://urldefense.com/v3/__http:/journals.plos.org/plosone/s/data-availability*loc-unacceptable-data-access-restrictions__;Iw!!PDiH4ENfjr2_Jw!GFqrB7o03x83g4bPbnNxGR7EdkciuYQkUswlPRyUblGIlgjuJ-4192X2h763iNcnsoCePBxSwcocpFfry3db2eGsIw$)**.**

**Before we proceed with your manuscript, please address the following prompts:**

a) **If there are ethical or legal restrictions on sharing a de-identified data set, please explain them in detail (e.g., data contain potentially identifying or sensitive patient information, data are owned by a third-party organization, etc.) and who has imposed them (e.g., a Research Ethics Committee or Institutional Review Board, etc.). Please also provide contact information for a data access committee, ethics committee, or other institutional body to which data requests may be sent.**

**b) If there are no restrictions, please upload the minimal anonymized data set necessary to replicate your study findings to a stable, public repository and provide us with the relevant URLs, DOIs, or accession numbers. For a list of recommended repositories, please see**

[**https://journals.plos.org/plosone/s/recommended-repositories [journals.plos.org]**](https://urldefense.com/v3/__https:/journals.plos.org/plosone/s/recommended-repositories__;!!PDiH4ENfjr2_Jw!GFqrB7o03x83g4bPbnNxGR7EdkciuYQkUswlPRyUblGIlgjuJ-4192X2h763iNcnsoCePBxSwcocpFfry3eE36bmGQ$)**. You also have the option of uploading the data as Supporting Information files, but we would recommend depositing data directly to a data repository if possible.**

**We will update your Data Availability statement on your behalf to reflect the information you provide.**

Due to the potentially identifiable nature of the qualitative transcripts, these data cannot be made publicly available presently. Data sharing is restricted by approvals University of Manchester [Ref 14566], Indonesia's National Research and Innovation Agency [Ref: 010/KE.03/SK/11/2022] and the Faculty of Nursing, the University of Indonesia [Ref KET-237/UN2.F12.D1.2.1/PPM.00.02/2022]. De-identified data will be deposited in an appropriate repository following publication of the primary study outputs, in line with the study’s ethical approvals. In the interim, relevant data are available upon reasonable request, subject to appropriate ethical and governance approvals, via the University of Manchester Information Governance Office (information.governance@manchester.ac.uk).

**7. If the reviewer comments include a recommendation to cite specific previously published works, please review and evaluate these publications to determine whether they are relevant and should be cited. There is no requirement to cite these works unless the editor has indicated otherwise.**

Noted.

**Reviewer 1:**

**General comments**

This manuscript addresses an important and timely topic on low-intensity psychological interventions (LIPIs). However, there are several major methodological and conceptual issues that need to be addressed before the manuscript can be considered for publication.

**Major comments**

1. **The scope of this paper focuses solely on *acceptability*. What was/were about the *feasibility* and *sustainability*?**

This paper focuses specifically on the acceptability of low-intensity psychological interventions, which represents a distinct and important dimension of implementation. While acceptability contributes to feasibility and may influence longer-term sustainability, it does not fully capture these broader constructs. A comprehensive assessment of feasibility and sustainability would require different data, including system-level perspectives (e.g. service planners, policymakers) and longitudinal evaluation of delivery and outcomes, which are beyond the scope of the current study. These aspects are being examined in parallel work within the wider programme in later work packages which are focused on implementation and sustainability and will be reported separately. To address this in the manuscript, we have clarified the scope to emphasise acceptability as a key, but not exhaustive, component of implementation (Page 29, paragraph 1).

2. **The term *low-intensity psychological interventions* should be clearly defined earlier in the Introduction (around line 68). A concise operational definition, including examples relevant to the Indonesian context, would improve readability and conceptual clarity. Also, the manuscript does not sufficiently discuss what kind of LIPI and how it would be delivered. In the Methods section, author should add amore details on the LIPI: who would deliver the intervention (and eligibility criteria), the dosage and intensity, that was intended by authors and asked to the participants. These are central to assessing acceptability and feasibility.**

We have revised the manuscript to improve clarity regarding the definition and description of low-intensity psychological interventions as suggested. In the Introduction, we now provide a clearer operational definition of LIPIs, including examples relevant to the study context and clearly differentiated from other forms of interventions such as counselling (Pages 4-5)

In the methods section, we have added further detail on the intervention model presented to participants, specifying that this involved a guided self-help approach incorporating behavioural activation, cognitive restructuring, worry management, and healthy lifestyle strategies, delivered over approximately 6–8 sessions by trained Kader. As this study explored the acceptability of a proposed intervention model rather than evaluating a standardised protocol, the intervention was described in sufficient detail to support informed discussion while retaining some flexibility (page 11).

3. **The inclusion criteria (Methods, line 164) are insufficiently specified. Criteria should be clearly defined for each stakeholder group, including:**
   - **Health workers (e.g., nurses, psychologists, psychiatrists, general practitioners)**
   - **Patients (e.g., setting: hospital vs. primary care; diagnostic status)**
   - **Family members (e.g., parents, spouses, caregivers)**
   - **Community leaders (clear definition and role)**
   - **Traditional healers (clear definition and scope of practice)**
   - **The use of “OR” in the inclusion criteria is inappropriate and should be revised.**

We have clarified the inclusion criteria to provide greater specificity across stakeholder groups. While a common set of overarching criteria was applied, we have revised the methods to more clearly indicate how these related to each group, including examples of health workers (e.g. nurses, psychologists, general practitioners), family members (e.g. spouses, caregivers), and community stakeholders (e.g. community leaders, traditional healers, third-sector professionals).We also clarify that participants with experience of anxiety and depression were recruited from primary care and community settings based on recent experience rather than formal diagnostic criteria. We have removed the OR in the inclusion criteria (Pages 9-10).

4. **The rationale for including traditional healers is unclear. Are they potential deliverers of LIPI, referral agents, or contextual stakeholders? This should be clearly explained.**

Traditional healers were included as key community stakeholders due to their important role in shaping help-seeking pathways and interpretations of mental distress within the Indonesian context. They were not considered primary deliverers of the low-intensity intervention, but were included to capture perspectives from those who influence community understanding, engagement, and potential referral pathways. We have clarified this rationale in the methods section to make their role within the study more explicit (Page 9).

5. **It is also unclear why representatives from district or provincial health offices (policy makers or programme planners) were not included, given their central role in implementation and sustainability. The authors should justify this omission or discuss it as a limitation.**

See response to point 1 above. The focus of this study was on the acceptability of low-intensity psychological interventions from the perspectives of those most directly involved in receiving, delivering, and shaping support at the community level. As such, we prioritised participants with lived experience, frontline providers, and key community stakeholders. Representatives from district or provincial health offices were not included, as their perspectives are more closely aligned with system-level planning, implementation, and sustainability considerations, which were beyond the scope of the current acceptability-focused study. We have clarified this in the manuscript and now acknowledge the absence of policy and programmatic perspectives as a limitation. We also note in the discussion that these broader system-level issues are being explored in parallel work within the wider programme (Page 34-35).

6. **The exclusion criteria are not clearly described. It should be specified for which stakeholder groups these criteria applied.**

We have now specified that stated exclusion criteria applied to people with experience of anxiety and depression in the methods section (Pages 9-10).

7. **The manuscript didn’t state if purposive sampling was used, and the specific criteria guiding purposive selection are not clearly explained. Please clarify how participants were selected and what characteristics were prioritized.**

This is currently stated in the manuscript in the methods under the sampling and recruitment section. Purposive sampling was used to ensure diversity in terms of area, gender, age and condition (Page 9).

8. **The description of recruitment raises several questions:**
   - **What registry or sampling frame was used to identify potential participants?**
   - **What was the response rate?**
   - **Were there refusals, and if so, what were the reasons?**
   - **How did the authors ensure that participants were appropriate representatives of their stakeholder groups?**

Following comments from other reviewers, we have clarified the recruitment process in the methods section to provide greater transparency regarding how participants were identified and selected. Participants were recruited through a combination of purposive and network-based sampling across study sites, including primary care services, community settings, and established professional and community contacts. As such, there was no single registry or sampling frame used, but rather a multi-source approach to ensure inclusion of relevant stakeholder groups. Given this recruitment strategy, formal response rates were not systematically recorded, and reasons for non-participation were not consistently captured across sites. We have clarified this in the manuscript. To ensure participants were appropriate representatives of their stakeholder groups, eligibility criteria were defined for each group, and purposive sampling was used to achieve diversity in terms of role, geography (urban/rural), gender, and experience. We have detail on this in the sampling and recruit section of the methods (page 8-9).

9. **The criteria for deciding whether participants joined in-depth interviews or focus group discussions are unclear. Please clarify how and why these different data collection methods were used.**

As outlined in the methods section, after consultation with local collaborators interviews were conducted with people with lived experience due to the sensitive nature of the topics discussed, allowing for more private and in-depth exploration of personal experiences. Focus group discussions were used with other stakeholder groups to facilitate shared discussion and capture both individual and collective perspectives. We have revised the manuscript to make this distinction more explicit and to clarify the reasoning behind the use of these complementary methods (Pages 10-11).

10. **Table 1 (Study locations). Several city names are listed, but the rationale for selecting these cities is not explained. Please justify the choice of study sites.**

We have clarified the rationale for the selection of study sites in the manuscript. The cities included were selected to reflect the diversity of contexts across Java, including variation in geographical location, urban and rural settings, and levels of health service provision (Page 7).

11. **Table 2 (Participant characteristics). Table 2 is confusing and would benefit from reorganisation. General characteristics (e.g., age, gender) should be separated from stakeholder-specific variables. This table, along with the accompanying text, should be moved from the Methods section to the Results section and the text should be substantially shortened.**

We have reviewed Table 2 and the accompanying text to improve clarity and readability. In particular, we have streamlined the presentation and reduced repetition in the accompanying description as recommended. We have retained Table 2 within the Methods section, as per the journal’s formatting conventions and to maintain a clear distinction between participant characteristics and study findings. However, we have ensured that the table presents general and stakeholder-specific characteristics more clearly to improve interpretability. We have also substantially shortened the narrative summary as requested (Pages 12-15).

12. **The study refers to seven components (*affective attitude, burden, ethicality, intervention coherence, opportunity costs, perceived effectiveness, and self-efficacy*) as the analytic framework. However, the Results section is not structured according to these components. The authors should explain this deviation or revise the Results to align clearly with the stated framework.**

As outlined in the methods section, the seven TFA constructs were used as the deductive analytic framework during indexing and charting, in line with framework analysis. The results are not organised as seven separate construct-based headings because the final stage of the framework analysis employed involves interpretive synthesis across constructs, allowing us to capture broader patterns, intersections, and contextual influences on acceptability. As clarified in the revised manuscript, this was a deliberate analytic choice rather than a deviation from the framework. TFA therefore structured the analysis, but the presentation of findings prioritised higher-order themes to avoid a purely descriptive mapping of data onto pre-specified domains (Pages 15-16).

13. **The manuscript does not clearly describe the interview or FGD guides used. Please specify:**
    - **What instruments were used**
    - **Whether they were adapted from existing tools**
    - **How their validity or credibility was ensured**

We have revised the manuscript (Page 11) to provide greater detail regarding the interview and focus group discussion guides. Topic guides were developed to explore participants’ perspectives on the acceptability of low-intensity psychological interventions and were broadly informed by the Theoretical Framework of Acceptability (TFA). The guides were developed in collaboration with local partners and refined through consultation with the research team and community advisors to ensure cultural relevance and appropriateness. As part of an iterative qualitative approach, the guides were also reviewed and adapted during data collection as needed.

14. **Lines 315–316: The statement that “most participants, particularly non-professionals, had limited awareness of mental health” appears misleading. Patients diagnosed with depression or anxiety would be expected to have some awareness of these conditions. The quoted data seem to reflect *health workers’ perceptions* of patients’ awareness rather than patients’ own views. This interpretation should be revised.**

Our intention was to highlight limited awareness of formal diagnostic terminology (e.g. anxiety and depression) across stakeholder groups, rather than a complete lack of awareness of mental distress among participants with lived experience. We have revised the manuscript to clarify that while participants recognised and described experiences of distress, there was limited use and understanding of clinical categories of anxiety and depression across groups (Pages 17-18).

15. **LIPI is a complex concept and likely unfamiliar to lay participants. The Methods section should clearly describe how LIPI was introduced during interviews, how understanding was checked, and how misunderstandings were addressed.**

We have now added this detail to the manuscript on pages 10-11.

16. **The manuscript implies that depression and anxiety lead to stigma. Conceptually, stigma is more appropriately framed as a *determinant* of mental health conditions. This relationship needs clarification.**

Our analysis conceptualises stigma as a contextual and social factor that shapes how mental distress is understood, disclosed, and responded to within communities, including influencing help-seeking behaviour and intervention acceptability. We have reviewed the manuscript to ensure this distinction is clearly articulated and have refined wording where necessary to avoid any potential ambiguity. This clarifies that stigma is treated as a broader social determinant of engagement with care, rather than an outcome of mental health conditions themselves.

17. **The quote stating that people with anxiety “don’t want anyone to know” and “try to look happy” appears to reflect misunderstanding of anxiety disorders. The authors should critically reassess whether this quote validly represents anxiety or reflects lay misinterpretation.**

Our intention was to illustrate how some stakeholders perceived anxiety-related distress to be hidden, difficult to identify, or deliberately concealed in community settings. We have revised the manuscript (Page 19) to clarify that this quotation represents a lay/stakeholder interpretation of how distress may be expressed or masked in practice.

18. **The quote describing people as “angry or emotionally unstable” lacks clarity. It is unclear whether respondents were referring to depression, anxiety, or other conditions. The authors should clarify this in the analysis and check the validity.**

This quotation reflects a stakeholder perspective on how mental distress is perceived and described in everyday language, including non-specific terms such as “angry” or “emotionally unstable.” We have revised the manuscript to clarify that this reflects lay conceptualisations of mental health problems, which often do not align neatly with diagnostic categories such as anxiety or depression. We have also adjusted the surrounding text to avoid over-attribution of the quote to specific conditions and instead present it as reflecting broader perceptions of mental distress and barriers to engagement with care.

19. **Although the study claims to explore acceptability of LIPI, many quotes and analyses do not clearly refer to LIPI itself. For example, statements such as “I think it is really very much needed” are vague, and it is unclear what “it” refers to. The analysis should more explicitly focus on LIPI.**

We have revised the results section to improve clarity by ensuring that the relevance of quotations to LIPI is more explicitly articulated in the accompanying analysis or within the quote itself.

**Minor comments**

1. **Please conduct a thorough grammar and language check, particularly ensuring consistent use of the past tense in the Methods section.**

We have conducted a full grammar and language check and ensured consistent use of past tense in the methods section.

2. **Abstract (line 37): Please spell out *LIPI* at first mention.**

We have checked and this was spelt out in full in the first instance in line 24.

3. **Check punctuation and consistency throughout the manuscript**

We have conducted a full grammar and language check and ensured consistent use of past tense in the methods section.

4. **The discussion of economic growth in the Introduction (lines 91–94) seems tangential. The Indonesian context should be introduced earlier and more directly linked to mental health system challenges.**

We have revised this paragraph of the introduction to more clearly link the discussion of economic context to mental health system challenges, including low health expenditure, workforce shortages, and limited access to care. In particular, we have tightened the wording to emphasise how these structural constraints underpin the need for scalable approaches such as low-intensity psychological interventions.

**Reviewer #2: The topic of this manuscript is important and show what really happen in reality especially in Indonesia setting. Mental health even it become one of the health priority in health ministry program, still the application especially in the community are still facing many challenges. Especially with lay workers or Kader-- they have significant role however, it's only their side job, they don't have well financial support and because it is voluntary, the Kader can be replaced over time, the same with nurses in community health centre, too many programs for one man job. I think, this paper should address more this aspect in discussion and also in future recommendations.**

We agree that the realities of workforce capacity and sustainability, particularly for Kader and primary care staff, are critical to the future use of low-intensity interventions in Indonesia. In response, we have strengthened the Discussion to more explicitly acknowledge the voluntary and often unpaid nature of Kader roles, the potential for workforce turnover, and the competing demands placed on community health staff and made recommendation for future research as recommended by reviewer 2. We also highlight the implications of these factors for intervention feasibility and sustainability, including the need for appropriate training, supervision, and financial and system-level support (Page 32).

**Reviewer #3: The manuscript addresses an important and timely topic: the acceptability of low-intensity psychological interventions in Indonesia, particularly in a context where depression and anxiety remain undertreated and where task-shifting through non-specialist workers may offer a scalable solution. The study is strengthened by its multi-stakeholder design, inclusion of patients, families, health workers, Kader, and community/religious leaders, and use of the Theoretical Framework of Acceptability. The sample is substantial for qualitative work, with 140 participants across 12 sites in Java.**

We thank reviewer 3 for this positive feedback.

**However, several areas require strengthening before publication.
First, the manuscript needs a clearer statement of its analytical contribution. The current framing is largely descriptive. The authors should explain more explicitly what this study adds beyond identifying barriers such as stigma, low literacy, privacy concerns, and workload. The strongest potential contribution is not simply that LIPIs are “acceptable,” but that acceptability in Indonesia is conditional, socially negotiated, and shaped by trust, privacy, gendered burden, religious/community legitimacy, and the perceived role of Kader.**

We have revised both the abstract and the Discussion to more explicitly foreground our conceptual contribution in line with this feedback. In the abstract (results section), we now emphasise that acceptability is not uniform but contingent on interacting social, cultural and structural factors. In the discussion, we have strengthened the opening paragraph to highlight that acceptability in this context is conditional and socially negotiated, rather than an inherent property of the intervention.

**Second, the concept of “acceptability” needs sharper theorisation. The manuscript uses the TFA well, but the findings suggest that several domains overlap, especially ethicality, affective attitude, perceived effectiveness, and intervention coherence. The authors should clarify how they distinguished these constructs during coding and interpretation. Otherwise, the analysis risks becoming a thematic mapping exercise rather than a theoretically informed qualitative analysis.**

This process is outlined in the methods section; however, in response to this feedback we have revised the manuscript to make this more explicit. As described, the research team collectively reviewed a subset of transcripts early in the analysis to operationalise the TFA constructs within the Indonesian context. This process was used to clarify how closely related domains (e.g. ethicality, affective attitude, perceived effectiveness, and intervention coherence) would be interpreted and applied, including identifying where constructs overlapped and how distinctions would be maintained during coding. Analysis was not limited to simple categorisation; rather, coding was undertaken iteratively, with attention to both within-construct variation and relationships across constructs. Overarching themes were subsequently developed across TFA domains to capture these intersections and avoid reducing findings to a purely descriptive mapping exercise. We have strengthened the description of this process across the methods and discussion to emphasise the theoretically informed nature of the analysis which we hope addresses this comment (Page 16).

**Third, the paper should engage more critically with the limitations of using the TFA in LMIC and collectivist contexts. The discussion begins to do this, noting that TFA was developed largely from high-income country literature and may underrepresent LMIC conceptualisations of acceptability. This is one of the most interesting parts of the paper and should be elevated earlier and developed more strongly as a key theoretical contribution.**

In line with this comment and the comment from reviewer 4 about the importance of this finding, we have moved this up in the discussion (first paragraph) to focus more prominently.

**Fourth, the manuscript should avoid overclaiming cultural generalisability. Data were collected only in Java, while Indonesia is highly diverse in ethnicity, religion, language, geography, and health system access. The limitation is acknowledged, but the title and conclusion sometimes imply broader Indonesian applicability. The authors should consistently specify “Java, Indonesia” or explain why findings may plausibly inform wider Indonesian implementation.**

Noted. We have ensured that the title and conclusion to specify Java, Indonesia.

**Fifth, the role of Kader requires deeper critical analysis. The manuscript reports high trust in Kader in rural areas but concerns in urban areas around confidentiality, gossip, and skills. This rural–urban contrast is highly important and should be treated as a central finding rather than a secondary observation. It has direct implications for implementation models: Kader-led delivery may not be equally acceptable across settings.**

We have strengthened the manuscript to foreground this more clearly in both the results and discussion. In particular, we now emphasise that acceptability of Kader-led delivery is context-dependent, with higher trust in rural settings and greater concerns around confidentiality, professionalism and skills in urban contexts. We also highlight the implications of this for future use of such interventions, noting that Kader-led models may require adaptation across settings and may not be uniformly acceptable. These revisions clarify the central role of this finding within the overall analysis (page 31-32)

**Sixth, the gender dimension should be strengthened. The finding that women may need permission from husbands and face household responsibilities that limit participation is important. This should be linked more explicitly to gendered autonomy, household decision-making, and mental health service access. At present, gender appears in the findings but is underdeveloped analytically – pull out gender a bit more in discussion.**

We have strengthened the discussion to more explicitly interpret these data in relation to gendered autonomy, household decision-making, and access to care as recommended. We now highlight how participation in the intervention was often mediated through household power dynamics and caregiving roles in the results and discussion, indicating that engagement is shaped not only by individual willingness but also by gendered social norms. We have also incorporated a brief reference to these dynamics in the abstract (results section) to ensure visibility.

**Seventh, the manuscript would benefit from a clearer implementation implications section. The findings point to several practical recommendations: community mental health literacy campaigns, confidentiality protocols for Kader, role clarification for religious/traditional healers, compensation or workload protection for Kader, flexible session timing, and privacy-sensitive delivery models. These should be presented in a concise table mapping barriers to adaptation strategies.**

While some practical implications can be drawn from the data, developing comprehensive and operationalised implementation recommendations would require input from a broader range of stakeholders (e.g. policymakers and service planners) and systematic co-design work, which is beyond the scope of the current study. We have; however, incorporated clearer narrative guidance on key areas for future work in the discussion, including mental health literacy, confidentiality, role clarity, and workforce support, and highlight that these are being explored further in ongoing work and hope this reassures the reviewer.

**Finally, some writing needs tightening. Several sentences are long, repetitive, or grammatically awkward. The abstract and conclusion should be more direct. For example, rather than saying the study shows “cultural, structural, and interpersonal factors shape intervention acceptability,” the authors could state: “LIPI acceptability in Java depends on whether interventions can be made private, trusted, culturally legitimate, low-burden, and clearly differentiated from general counselling.”**

In line with this comment and feedback from other reviewers, we have revised the text throughout to reduce repetition, simplify sentence structure, and improve overall readability. We have also strengthened the abstract and conclusion sections in line with this specific comment to make the key findings more direct and specific.

**Reviewer #4: The paper entitled “Stakeholder perspectives on the acceptability of low-intensity psychological interventions in Indonesia: A theoretically framed qualitative study” addresses an important and timely topic in global mental health and implementation research. The manuscript is generally well written, methodologically sound, and draws on a large and diverse qualitative dataset. However, there are several areas that require clarification, tightening, and further conceptual refinement before the manuscript is suitable for publication. These are outlined below.**

We thank reviewer 4 for this positive feedback.

**Abstract**

**• Page 2, Lines 24 to 25: The sentence “Low-intensity psychological interventions, delivered by trained non-specialists offer a promising pathway…” requires a comma after “non-specialists.”** Comma added.

**• Page 2, Lines 36 to 42: The Results section is quite dense and introduces multiple TFA constructs simultaneously. Consider simplifying the wording and reducing conceptual repetition to improve readability.** We have revised the results section to reduce conceptual repetition and improve readability as recommended.

**• Page 3, Lines 44 to 48: The claim that this study is “one of the first theoretically framed qualitative explorations” may be somewhat overstated unless supported by clearer positioning within the existing literature. Please moderate this statement.** We have moderated this sentence as follows to ensure the focus is on acceptability too: *This study provides a theoretically informed qualitative exploration of LIPI acceptability in Southeast Asia, highlighting culturally relevant factors shaping its acceptability.*

**Introduction**

**• Page 4, Lines 52 to 64: The opening paragraph is overly dense and attempts to cover prevalence, economic burden, workforce shortages, and treatment gaps simultaneously. Consider breaking this into shorter and more focused paragraphs.** We have now split the opening paragraph in the introduction into three shorter, more focused paragraphs as recommended by reviewer 4.

**• Page 4, Lines 61 to 62: The statement that the ratio of therapists in LMICs is “approximately 0.5% that of high-income countries” is difficult to interpret as currently written. Please revise for clarity.**

We have revised this sentence for clarity: *The availability of mental health therapists per 100,000 population in LMICs is extremely limited—approximately 200 times lower than in high-income countries*

**• Page 5, Lines 82 to 87: The discussion on low-intensity interventions and task shifting is useful. However, it would strengthen the paper to briefly distinguish low-intensity interventions from general counselling earlier in the manuscript, especially given that confusion around this issue later becomes a major finding.**

We have now added a sentence to distinguish between low-intensity psychological interventions and general counselling in order to address this comment. *Such approaches are structured and protocol-driven, in contrast to the more open-ended and non-directive nature of general counselling.*

**• Page 6, Lines 100 to 109: The rationale for conducting this study in Indonesia is clear. However, the discussion on Muslim-majority settings, sociocultural norms, and implementation gaps could be better integrated conceptually rather than presented as separate issues.**

We have revised the two paragraphs reviewer 4 refers to in order to better integrate Muslim-majority settings, sociocultural norms and implemetantion gaps rather than presenting them as separate issues (Page 6).

**Materials and Methods**

**• Page 7, Lines 125 to 126: The authors state that a qualitative descriptive design was used. Please provide a brief rationale for why this design was selected over other qualitative approaches such as phenomenology, grounded theory, or reflexive thematic analysis.**

We have now added a brief rationale to the methods section to justify the use of qualitative descriptive design (Page 7).

**• Page 8, Lines 160 to 162: The manuscript states that data collection continued until “data saturation was achieved.” Please explain how saturation was operationalised and determined, particularly across the different stakeholder groups.**

We have now included our definition of data saturation and how this was operationalised and determined across different stakeholder groups (page 9).

**• Page 8, Lines 167 to 168: It is unclear how participants with anxiety and depression were identified. Please clarify whether these were clinically diagnosed conditions, self-reported experiences, screening-based identifications, or based on prior service contact. – put in limitations about screening.**

We have now included additional detail to specify that participants were recruited through professional judgement/primary care records and included a reflection in the strength and limitations section about this impact this may have had on the results (Page 10).

**• Page 9, Lines 184 to 188: The recruitment strategy for community stakeholders appears broad and heterogeneous. Please clarify whether any effort was made to ensure balance across stakeholder categories such as traditional healers, religious leaders, policymakers, and community leaders.**

We have now clarified in the manuscript that we used purposive sampling to ensure representation across key community stakeholder groups, including spiritual, religious and community leaders, and traditional healers. Recruitment was iteratively monitored to capture a breadth of perspectives across these categories, although formal quotas were not specified (Page 9).

**• Page 10, Lines 201 to 202: The sentence “focus group discussions last just between 60 and 70 minutes” requires grammatical revision.**

Changed.

**• Page 10, Lines 211 to 212: Only 5% of transcripts were back translated. Please provide a brief justification for why this proportion was considered adequate for ensuring translation accuracy.**

We have now made it explicit that this proportion of transcripts were back-translated following the guidance by Twinn et al who advocates for selective back translation as a quality check rather than a requirement for all transcripts (Page 12).

**Data Analysis**

**• Page 15, Lines 278 to 293: The analysis section is generally strong. However, there appears to be some conceptual stretching of the TFA constructs. For example, “perceived need” is incorporated under affective attitude, and “collective ethicality” is introduced as an extension of the framework. Please clarify more explicitly which findings emerged inductively beyond the original TFA framework rather than retrospectively fitting all findings into TFA domains.**

We agree that greater clarity was needed regarding the analytic approach. As described in the revised manuscript, the research team collectively reviewed a subset of transcripts early in the analysis to consider the cultural applicability of TFA constructs within the Indonesian context and to operationalise their use. This process informed how constructs were interpreted and applied, including distinguishing between individual and collective dimensions of ethicality, rather than redefining the framework itself. Analysis was therefore not purely deductive; we combined TFA-informed coding with inductive coding to capture data that did not align neatly with existing constructs. Overarching themes were developed across and within TFA domains to reflect relationships between constructs and context-specific influences on acceptability. Concepts such as perceived need and collective ethicality are now more clearly presented as inductively derived, context-specific interpretive insights used to understand acceptability in this setting, rather than as formal extensions of the TFA. We have revised the manuscript to make this analytic process and distinction more explicit and hope this addresses this important comment (Pages 16-17).

**• Page 15, Lines 281 to 282: There is a typographical error in the phrase “ethicality and and agreeing.”** Revised.

**• Page 15, Lines 286 to 286: The statement regarding data “that fell out with the TFA framework” would benefit from further clarification. How were these inductive themes ultimately integrated into the analysis?** See comment above which now addresses this comment.

**Results**

**• Page 16, Lines 313 to 322: The findings regarding mental health literacy and stigma are important. However, similar points recur repeatedly across later sections. The Results section would benefit from further condensation and synthesis to reduce repetition.** We have reviewed the results section carefully and removed repetition to condense where possible.

**• Page 16, Lines 317 to 319: The explanation of local terminology relating to mental illness is useful, though the phrase “people with psychological problem who are risk of developing mental health disorders” requires grammatical revision.** Revised.

**• Page 17, Lines 338 to 343: The confusion between LIPIs and counselling appears to be one of the manuscript’s most important findings. It may be useful to expand the interpretation of this issue rather than treating it primarily as low intervention coherence.**

We agree and have now clarified that the implications of this finding extend beyond intervention coherence (Page 29-30) and also picked up on the broader implications for fidelity, engagement and outcomes in the discussion which we hope addresses this point.

**• Page 18 to 22: Several quotations are quite long and occasionally difficult to follow due to translation structure. Some quotations could be shortened without losing conceptual meaning.**

We have reviewed quotations in the results sections and shortened where possible without losing conceptual meaning.

**• Page 19, Lines 393 to 399: The discussion around self-reliance, autonomy, and social isolation is interesting and could be analysed more deeply in relation to Indonesian sociocultural norms.**

We have now expanded on this discussion to address this point and incorporate (). We have expanded the discussion to more explicitly situate perceptions of self-reliance and autonomy within Indonesian sociocultural norms. In particular, we now highlight how the perceived acceptability of self-help may be shaped by contextual factors such as stigma, concerns about burdening others, and the central role of family and community networks in support-seeking. The revised text also reflects how autonomy is understood in a more relational way in this context, with participants viewing self-help approaches as complementary to, rather than replacing, existing social support structures. This has strengthened our interpretation of how cultural norms influence the acceptability of low-intensity interventions (Page 21 & 30).

**• Page 21, Lines 443 to 450: The role of religious and traditional healers is highly relevant. However, the analysis remains somewhat descriptive. It would strengthen the paper to discuss more critically how traditional and biomedical understandings of mental distress intersect or potentially conflict.**

We have expanded this section to more explicitly explore how traditional, religious and biomedical understandings of psychological distress may intersect. In particular, we now highlighted that while apparent alignment with religious and communal values may support acceptability, participants’ interpretations of the intervention may be shaped by existing spiritual and social explanatory frameworks. This has important implications for how LIPIs are understood and delivered in practice, and we have clarified this more explicitly in the revised manuscript in both the results and discussion (Page 22 & 32).

**• Page 22 to 24: The findings relating to stigma, privacy, and confidentiality are among the strongest parts of the manuscript. However, there is considerable overlap between discussions of ethicality, stigma, trust, and social judgement. Consider streamlining these sections conceptually.** In line with the comments above, we have reviewed our results section and streamlined sections reducing repetition where possible.

**• Page 25, Lines 529 to 534: The urban versus rural differences in trust toward Kader are particularly important findings. These distinctions deserve greater analytic attention in both the Results and Discussion sections.** We agree that the urban–rural differences in trust towards Kader are an important finding. We have strengthened the results section to make these distinctions clearer and have expanded the results and discussion to highlight their potential implications. In particular, we now emphasise how contextual factors such as community familiarity, expectations of confidentiality, and perceptions of professional expertise may shape trust in Kader across settings, and note this as an area requiring further investigation so as not to extend past the scope of our data (Pages 25-26).

**• Page 26, Lines 549 to 589: The discussion of burden and opportunity costs is valuable, especially the gendered dimensions of participation. However, some sections become repetitive and could be tightened. –** See above response about reviewing and condensing the results section to remove repetition.

**Discussion**

**• Page 28, Lines 600 to 609: The opening paragraph of the Discussion provides a good summary. However, several points raised here are repeated multiple times later in the section.**

In line with comments from reviewers 1-4, we have reviewed the manuscript as a whole to reduce duplication.

**• Page 29, Lines 634 to 646: The discussion around affective attitude and cultural value alignment is interesting. However, the manuscript would benefit from a deeper explanation of how Islamic, communal, and gender norms specifically shape intervention acceptability.**

We have expanded this section to more explicitly describe how these norms influence perceptions of the intervention. In particular, we now highlight how alignment with religious values (e.g. responsibility and holistic wellbeing), communal expectations around maintaining social harmony, and gendered norms around acceptable forms of help-seeking contribute to positive affective responses (Page 30-31).

**• Page 30, Lines 648 to 660: The discussion around stigma and confidentiality is useful. However, the phrase “the modality of the intervention” conflicting with norms of discretion could be explained more clearly with practical examples.**

We have now revised this section to include practical examples of how delivery approaches (e.g. repeated home visits) may increase visibility within communities and therefore conflict with norms of discretion. This clarifies how concerns about confidentiality and social judgement may arise not only from who delivers the intervention, but from how it is delivered in practice (Pages 31-32).

**• Page 31 to 32, Lines 681 to 702: This is arguably one of the strongest sections of the manuscript. The reflection on the applicability of TFA constructs in LMIC settings is thoughtful and theoretically important. Consider foregrounding some of these conceptual reflections earlier in the paper.**

We have revised the discussion to foreground these conceptual insights earlier (in opening discussion paragraph), ensuring that key reflections on how TFA constructs operate and require interpretation within LMIC settings are introduced more prominently. This improves the visibility of the paper’s theoretical contribution while maintaining the overall structure of the discussion.

**Strengths and Limitations**

**• Page 32 to 33, Lines 704 to 724: The limitations section is appropriately balanced and realistic. The acknowledgment that most participants were recruited through health services is particularly important.** Noted.

**• Page 33, Lines 719 to 724: The reflections on translation challenges are appreciated. It may be helpful to briefly indicate whether these challenges materially influenced interpretation of any themes.**

We have clarified this point in the manuscript by indicating that whilst challenges arose in translating culturally specific terms, these were addressed through team discussion and by revisiting the original Bahasa Indonesia transcripts. This allowed meanings to be cross-checked where necessary, and we have now specified that these issues did not materially influence the interpretation of the key themes (Pages 34-35).

**Conclusion**

**• Page 33 to 34, Lines 735 to 742: The conclusion is generally appropriate and grounded in the findings. However, phrases such as “novel insights” and “contextually tailored, culturally congruent” are repeated frequently throughout the manuscript and could be simplified for conciseness.**

We agree that some phrases were repeated more frequently than necessary. We have revised the manuscript to improve conciseness by reducing repetition of phrases such as “novel insights” and “contextually tailored, culturally congruent,” and simplifying the language throughout, particularly in the conclusion.

We hope that the revised manuscript and our responses satisfactorily address the reviewers’ comments. We are grateful for the opportunity to revise this work and appreciate your further consideration of our manuscript.

Yours sincerely,

Professor Helen Brooks

Corresponding author on behalf of the authorship team
