# Supplementary material for: Stakeholder perspectives on the anticipated acceptability of low-intensity psychological interventions in Java, Indonesia: A theoretically framed qualitative study
Source: PLoS One. 2026 Sep 29;21(9):e0358869. doi: 10.1371/journal.pone.0358869 (PMC13623157; doi:10.1371/journal.pone.0358869)
Supplement: Supplementary file 4 [file pone.0358869.s005.docx]

**Dear Editor,**

**RE: PONE-D-25-65667R1 - Stakeholder perspectives on the acceptability of low-intensity psychological interventions in Java, Indonesia: A theoretically framed qualitative study.**

Thank you for your email dated 19^th^ of October and for the detailed and constructive feedback. We have carefully revised the manuscript in response to all comments and believe the paper has been substantially strengthened as a result. We note that Reviewer 4 indicated that all previous concerns had been satisfactorily addressed. Reviewers 1 and 2 raised no substantive concerns in the previous review round and supported publication following revision.

We have also made extensive revisions in response to Reviewer 3's comments, including clarifying the distinction between anticipated and experienced acceptability, strengthening discussion of intervention coherence, moderating implementation-related claims, and expanding consideration of methodological limitations.

We recognise that Reviewer 3 raises broader concerns regarding the appropriateness of assessing acceptability prior to intervention delivery. Whilst we have sought to address these concerns throughout the manuscript, we believe some aspects reflect a conceptual difference regarding the role of prospective acceptability research within intervention development and implementation science. Our position is that examining anticipated acceptability prior to implementation is both consistent with the Theoretical Framework of Acceptability and an important component of cultural adaptation and implementation planning, particularly within LMIC settings where substantial formative work is recommended prior to intervention delivery.

We would also note that several of Reviewer 3's concerns appear to arise from expectations more closely aligned with post-implementation evaluation studies than with the aims of a qualitative descriptive study examining anticipated acceptability prior to implementation. Whilst we have revised the manuscript extensively in response to these comments, some concerns reflect the inherent boundaries of the study design rather than methodological shortcomings. The study was intentionally undertaken prior to implementation to identify cultural adaptation priorities, anticipated barriers, and stakeholder perspectives in the specific contexts where future delivery was planned. Importantly, the wider programme of work has subsequently completed implementation and evaluation activities; however, these address different research questions and were not the focus of the present study. We therefore hope the manuscript will be evaluated in relation to its stated objectives and contribution as a formative study informing intervention adaptation and implementation planning.

We therefore hope the editor will consider the revisions made and the extent to which the remaining concerns reflect differing interpretations of prospective acceptability research rather than methodological limitations that can be fully resolved within the current study design.

Response to Reviewer 3

**Comment 1: The study claims to assess “acceptability,” but participants had not actually experienced the intervention This is the most fundamental concern. The study was conducted before implementation of the LIPI, specifically to inform cultural adaptation. Thus, participants were evaluating a proposed intervention model, rather than an intervention they had actually received or delivered. The authors themselves acknowledge elsewhere in the response that the intervention presented to participants was not yet a standardized protocol but a proposed guided self-help model of approximately 6–8 sessions delivered by Kader. Consequently, much of what is described as “acceptability” is more accurately anticipated or hypothetical acceptability. This distinction is critical under the TFA because prospective perceptions may differ substantially from experienced acceptability once participants encounter the actual intervention burden, therapeutic relationship, confidentiality arrangements, Kader competence, session intensity, and treatment outcomes.
Yet the manuscript makes statements such as: “LIPI acceptability in Java depends on whether interventions are perceived as private, trustworthy, culturally legitimate, and feasible...”
and discusses “potential for uptake.” Without actual intervention exposure, these conclusions are stronger than the design permits. This is not readily fixed through textual revision. The central outcome in the title, objectives, analysis and conclusions is acceptability, while the empirical data primarily capture expectations about a hypothetical intervention.**

Response - We would respectfully note that the purpose of the study was not to evaluate experienced acceptability following intervention exposure, but to explore anticipated acceptability prior to implementation in order to inform cultural adaptation and implementation planning. This approach is consistent with the prospective application of the TFA and reflects the study objectives from the outset. The wider programme has subsequently completed implementation and evaluation activities, however these data were not available at the time this formative work was undertaken and address a different research question.

We therefore agree that participants were evaluating a proposed intervention model rather than one they had directly experienced. However, this was the explicit purpose of the study, which was undertaken prior to implementation to inform cultural adaptation and implementation planning. Consistent with the Theoretical Framework of Acceptability, we conceptualise acceptability as a construct that can be explored prospectively as well as retrospectively.

To avoid any potential overstatement, we have revised the manuscript throughout to more clearly distinguish anticipated acceptability from experienced acceptability and have strengthened discussion of this distinction within the limitations section. We have also moderated language relating to future uptake and implementation where appropriate.

For example, we have changed acceptability to anticipated or prospective acceptability throughout the manuscript including the title and abstract and ensured the limitations of such an approach are adequately reflected in the discussion.

**Comment 2 -  There is a serious construct-validity problem because many participants did not clearly understand what a LIPI was. The manuscript identifies low intervention coherence as a major finding: participants frequently conflated guided self-help/LIPIs with counselling. This creates a circular methodological problem. If participants did not adequately distinguish the intervention from counselling, it becomes unclear what intervention they were actually judging when answering questions about acceptability. Their positive attitudes, perceived effectiveness, burden, ethicality, or preferences may refer partly to counselling, general mental-health support, or simply receiving additional help—not specifically to the proposed LIPI. The problem becomes particularly apparent when the authors report that participants expressed “hope, optimism, and acceptance” toward the intervention despite limited understanding of it. This undermines the interpretability of several TFA domains. For example, it is difficult to establish meaningful perceived effectiveness for an intervention whose mechanism, intensity, provider role, and distinction from existing counselling were not consistently understood. Low intervention coherence is presented as a substantive result, but it simultaneously threatens the validity of the entire acceptability assessment. The manuscript does not sufficiently separate “acceptability of receiving mental-health support” from “acceptability of this specific LIPI model.”**

Response - We agree that limited intervention coherence creates challenges for interpretation. However, identifying misunderstandings regarding the nature, purpose and delivery of the proposed intervention was itself a central objective of this formative study. The study was explicitly designed to identify barriers to future implementation and adaptation requirements prior to delivery. The finding that participants frequently conflated low-intensity psychological interventions with counselling highlighted an important adaptation and implementation challenge that required addressing before implementation. Identifying such misunderstandings was therefore a central objective of the study rather than solely an unintended limitation. We have, however, strengthened the discussion to more clearly distinguish anticipated acceptability of receiving mental health support generally from anticipated acceptability of the proposed low-intensity intervention specifically, and expanded consideration of the implications of low intervention coherence for interpretation of the findings. We have added the following text to the Discussion:

***Whilst limited intervention coherence constrains interpretation of some acceptability domains, it also demonstrates the value of undertaking prospective acceptability work prior to implementation, as these misunderstandings may not otherwise have been identified until intervention delivery had already commenced. The findings therefore highlight important areas requiring clarification during subsequent adaptation, training and implementation activities.***

We would also note that, following early data collection activities, researchers introduced a standardised description of the proposed intervention at the beginning of interviews and focus groups to support participant understanding and facilitate discussion. Despite this, intervention coherence remained limited across stakeholder groups, further highlighting the importance of this finding and the value of identifying such misunderstandings prior to implementation. We have now clarified the methods section to explain how the proposed intervention was introduced during data collection and how researchers responded to early observations regarding intervention coherence.

**Comment 3: The sampling strategy creates substantial selection bias and limits the claimed community perspective. Recruitment relied on purposive and network-based sampling through primary-care services, community organisations and professional contacts, and response rates were not systematically recorded. More importantly, individuals with anxiety/depression were identified through primary-care professionals rather than standardized diagnostic procedures. The manuscript acknowledges that: “Patients were identified via primary care professionals as having depression and/or anxiety rather than formal diagnostic screening procedures.”
This is especially problematic for a study whose central themes include stigma, low mental-health literacy, delayed help-seeking, and barriers to accessing services. People already visible to health/community networks are likely systematically different from precisely those populations most affected by stigma, mistrust, social isolation, or avoidance of formal services.
The exclusion of individuals with recent/current suicide risk and some other conditions further means that the findings cannot readily represent people with more complex or severe needs.
The sampling mechanism potentially selects participants who are comparatively connected to health/community systems and receptive to intervention. This may partly explain the reported “broad consensus” and positive attitudes and creates an important threat to the central conclusions about community acceptability.**

Response – The large sample and multi-site design were intentionally used to maximise diversity of stakeholder perspectives across implementation contexts rather than to support theory generation or population-level inference. Consistent with qualitative descriptive methodology, our aim was to provide a rich and contextually grounded understanding of anticipated acceptability and identify adaptation priorities prior to implementation.

The exclusion of participants experiencing current suicide risk was required by the ethical approvals governing the study and reflected concerns regarding participant safety within a study that was not designed to provide clinical intervention.

We have nevertheless tried to reflect reviewer 3’s comment in the strengths and limitations section of the discussion:

***Participants were recruited primarily through existing healthcare and community networks and may therefore differ from individuals who remain socially isolated, highly stigmatised, disconnected from services, or otherwise difficult to reach. Findings should therefore be interpreted as reflecting a broad range of stakeholder perspectives rather than population-level views.***

The study did not aim to establish clinical diagnoses or compare diagnostic groups. Participants were recruited on the basis of lived experience of anxiety and depression within community settings, consistent with the exploratory aims of the study and the focus on stakeholder perspectives regarding anticipated acceptability. We have now clarified this within the methods section of the manuscript.

**Comment 4: The TFA appears to structure rather than genuinely deepen the analysis, producing a largely descriptive study
Despite being presented as a “theoretically framed” study, the analytical contribution remains limited. Indeed, an earlier reviewer identified exactly this problem, describing the manuscript as “largely descriptive” and noting that barriers such as stigma, low literacy, privacy concerns, and workload needed a clearer analytical contribution.
The revision reframes the contribution as acceptability being “conditional and socially negotiated,” shaped by trust, privacy, gendered burden and community legitimacy. However, much of the analysis still maps relatively predictable findings onto TFA categories—intervention coherence, affective attitude, ethicality, burden, perceived effectiveness, etc.—rather than demonstrating how the empirical findings extend, challenge, or substantially refine the TFA.
There is also conceptual overlap among domains. For example, privacy and stigma influence ethicality, affective attitude and willingness to participate; trust affects perceived effectiveness and acceptability; and cultural/religious legitimacy cuts across several constructs. This raises the possibility that the framework has imposed categorical organization on phenomena that are inherently relational.
The authors themselves claim that the findings show the need for “contextual interpretation” of TFA in LMIC settings, but the manuscript does not develop this far enough into a theoretically distinctive model or proposition.
For a very large qualitative dataset involving 140 participants and 12 sites, the analytical return is surprisingly modest. The paper provides useful descriptive implementation information, but the theoretical contribution does not appear proportionate to the scale of the study or sufficiently novel to overcome the other design limitations.**

Response – We explicitly set the study up using a qualitative descriptive design rather than a theory-generating methodology in order to address the stated aims. The intention was not to develop a novel theory of acceptability but to use the TFA as a sensitising framework to understand stakeholder perspectives and identify adaptation priorities within a qualitative descriptive study. Nevertheless, we have strengthened the discussion to articulate more clearly how our findings extend existing applications of the TFA in LMIC settings. Specifically, our findings suggest that acceptability in this context is not solely an individual cognitive judgement but is socially negotiated through relationships, trust, privacy concerns, family dynamics, community legitimacy and cultural norms.

We have also expanded discussion of how our findings relate to and extend existing work applying TFA within LMIC settings.

We have now added a synthesis paragraph to the discussion to highlight theoretical contributions:

***Across domains, acceptability appeared less as an individual cognitive judgement and more as a socially negotiated process shaped by trust, privacy, family relationships, community legitimacy and cultural expectations. This suggests that the acceptability of psychological interventions in LMIC settings may be understood not solely through individual perceptions of intervention attributes but also through the social and relational contexts in which intervention decisions are made.***

**Comment 5 - 5. Several implementation conclusions extend beyond what an acceptability study can establish
The manuscript repeatedly moves from participants' perceptions toward claims concerning implementation, fidelity, engagement, scalability and future uptake. For example, it argues that misunderstanding the intervention may lead to future deviations in delivery style and intensity and recommends training, supervision and monitoring to support fidelity.
Similarly, the discussion interprets perceived need as a “powerful motivator” capable of overriding low literacy and intervention-coherence barriers and suggests strong demand/openness across groups.
But no intervention was implemented, so the study did not observe actual:
uptake → engagement → adherence → fidelity → effectiveness → retention → sustainability.
Indeed, the authors explicitly acknowledge that feasibility and sustainability are outside this study and are being examined in later work packages. This makes some of the implementation-oriented interpretations premature.
This issue is particularly important given the authors' own finding that Kader already face heavy workloads and nurses anticipate difficulties providing supervision and training because of staff shortages. Positive hypothetical attitudes therefore cannot establish that the intervention will be acceptable when those constraints become real during delivery.
The conclusions occasionally cross the boundary between anticipated acceptability and evidence about actual implementation. Addressing this adequately would require post-implementation data rather than simply further discussion.**

Response - We agree that implementation outcomes cannot be inferred directly from anticipated acceptability data. Our intention was not to claim future implementation success but rather to identify factors that stakeholders perceived as likely to influence future delivery and engagement. We have revised the manuscript throughout to ensure this distinction is explicit and to avoid implying actual uptake, engagement, fidelity or sustainability and instead refer to participants' perceptions regarding the likely acceptability and future use of the intervention.

We have also revised several statements across the entire manuscript to ensure that references to uptake, engagement and implementation remain clearly framed as stakeholder perceptions rather than observed outcomes.

We hope these revisions satisfactorily address the remaining concerns. Should you require any additional information, please do not hesitate to contact me.

Professor Helen Brooks

***Corresponding Author on behalf of the authorship team.***
