# Supplementary material for: Stakeholder perspectives on the anticipated acceptability of low-intensity psychological interventions in Java, Indonesia: A theoretically framed qualitative study
Source: PLoS One. 2026 Sep 29;21(9):e0358869. doi: 10.1371/journal.pone.0358869 (PMC13623157; doi:10.1371/journal.pone.0358869)
Supplement: Peer review history

# Peer review: Decision Letter 0

**DOI:** 10.1371/journal.pone.0358869.r001

**Authors:** Kristiawan Indriyanto

29 May 2026

Dear Dr. Brooks,

Thank you for submitting your manuscript to PLOS ONE. After careful consideration, we feel that it has merit but does not fully meet PLOS ONE’s publication criteria as it currently stands. Therefore, we invite you to submit a revised version of the manuscript that addresses the points raised during the review process.

Please submit your revised manuscript by Jul 13 2026 11:59PM. If you will need more time than this to complete your revisions, please reply to this message or contact the journal office at plosone@plos.org. When you're ready to submit your revision, log on to https://www.editorialmanager.com/pone/ and select the 'Submissions Needing Revision' folder to locate your manuscript file.

- A letter that responds to each point raised by the academic editor and reviewer(s). You should upload this letter as a separate file labeled 'Response to Reviewers'.
- A marked-up copy of your manuscript that highlights changes made to the original version. You should upload this as a separate file labeled 'Revised Manuscript with Track Changes'.
- An unmarked version of your revised paper without tracked changes. You should upload this as a separate file labeled 'Manuscript'.

If you would like to make changes to your financial disclosure, please include your updated statement in your cover letter. Guidelines for resubmitting your figure files are available below the reviewer comments at the end of this letter.

If applicable, we recommend that you deposit your laboratory protocols in protocols.io to enhance the reproducibility of your results. Protocols.io assigns your protocol its own identifier (DOI) so that it can be cited independently in the future. For instructions see: https://journals.plos.org/plosone/s/submission-guidelines#loc-laboratory-protocols. Additionally, PLOS ONE offers an option for publishing peer-reviewed Lab Protocol articles, which describe protocols hosted on protocols.io. Read more information on sharing protocols at https://plos.org/protocols?utm_medium=editorial-email&utm_source=authorletters&utm_campaign=protocols.

As the corresponding author, your ORCID iD is verified in the submission system and will appear in the published article. PLOS supports the use of ORCID, and we encourage all coauthors to register for an ORCID iD and use it as well. Please encourage your coauthors to verify their ORCID iD within the submission system before final acceptance, as unverified ORCID iDs will not appear in the published article. *Only*  the individual author can complete the verification step; PLOS staff *cannot*  verify ORCID iDs on behalf of authors.

We look forward to receiving your revised manuscript.

Kind regards,

Kristiawan Indriyanto

Academic Editor

PLOS One

Journal Requirements:

When submitting your revision, we need you to address these additional requirements.

1.Please ensure that your manuscript meets PLOS ONE’s style requirements, including those for file naming. The PLOS ONE style templates can be found at

https://journals.plos.org/plosone/s/file?id=wjVg/PLOSOne_formatting_sample_main_body.pdf and

https://journals.plos.org/plosone/s/file?id=ba62/PLOSOne_formatting_sample_title_authors_affiliations.pdf

2. Please include a complete copy of PLOS’ questionnaire on inclusivity in global research in your revised manuscript. Our policy for research in this area aims to improve transparency in the reporting of research performed outside of researchers’ own country or community. The policy applies to researchers who have travelled to a different country to conduct research, research with Indigenous populations or their lands, and research on cultural artefacts. The questionnaire can also be requested at the journal’s discretion for any other submissions, even if these conditions are not met.  Please find more information on the policy and a link to download a blank copy of the questionnaire here: https://journals.plos.org/plosone/s/best-practices-in-research-reporting. Please upload a completed version of your questionnaire as Supporting Information when you resubmit your manuscript.

3. Please note that funding information should not appear in any section or other areas of your manuscript. We will only publish funding information present in the Funding Statement section of the online submission form. Please remove any funding-related text from the manuscript.

4. Thank you for stating the following financial disclosure:

“This research was funded by the NIHR [Global Health Research for Sustainable Care for anxiety and depression in Indonesia (Award ID NIHR134638)] using UK international development funding from the UK Government to support global health research. The views expressed in this publication are those of the author(s) and not necessarily those of the NIHR or the UK government.”

Please state what role the funders took in the study.  If the funders had no role, please state: "The funders had no role in study design, data collection and analysis, decision to publish, or preparation of the manuscript."

If this statement is not correct you must amend it as needed.

Please include this amended Role of Funder statement in your cover letter; we will change the online submission form on your behalf.

5. Please expand the acronym “NIHR” (as indicated in your financial disclosure) so that it states the name of your funders in full.

This information should be included in your cover letter; we will change the online submission form on your behalf.

6. We note that you have indicated that there are restrictions to data sharing for this study. PLOS only allows data to be available upon request if there are legal or ethical restrictions on sharing data publicly. For more information on unacceptable data access restrictions, please see http://journals.plos.org/plosone/s/data-availability#loc-unacceptable-data-access-restrictions.

Before we proceed with your manuscript, please address the following prompts:

a) If there are ethical or legal restrictions on sharing a de-identified data set, please explain them in detail (e.g., data contain potentially identifying or sensitive patient information, data are owned by a third-party organization, etc.) and who has imposed them (e.g., a Research Ethics Committee or Institutional Review Board, etc.). Please also provide contact information for a data access committee, ethics committee, or other institutional body to which data requests may be sent.

b) If there are no restrictions, please upload the minimal anonymized data set necessary to replicate your study findings to a stable, public repository and provide us with the relevant URLs, DOIs, or accession numbers. For a list of recommended repositories, please see

https://journals.plos.org/plosone/s/recommended-repositories. You also have the option of uploading the data as Supporting Information files, but we would recommend depositing data directly to a data repository if possible.

We will update your Data Availability statement on your behalf to reflect the information you provide.

7. If the reviewer comments include a recommendation to cite specific previously published works, please review and evaluate these publications to determine whether they are relevant and should be cited. There is no requirement to cite these works unless the editor has indicated otherwise.

Additional Editor Comments:

Please revise your paper in accordance with the reviewers' comments

[Note: HTML markup is below. Please do not edit.]

Reviewers' comments:

Reviewer’s Responses to Questions

**Comments to the Author**

1. Is the manuscript technically sound, and do the data support the conclusions?

Reviewer #1: Partly

Reviewer #2: Yes

Reviewer #3: Partly

Reviewer #4: Yes

**********

2. Has the statistical analysis been performed appropriately and rigorously?

Reviewer #1: N/A

Reviewer #2: N/A

Reviewer #3: N/A

Reviewer #4: N/A

**********

3. Have the authors made all data underlying the findings in their manuscript fully available?

The PLOS Data policy

Reviewer #1: No

Reviewer #2: Yes

Reviewer #3: No

Reviewer #4: Yes

**********

4. Is the manuscript presented in an intelligible fashion and written in standard English?

Reviewer #1: No

Reviewer #2: Yes

Reviewer #3: Yes

Reviewer #4: Yes

**********

Reviewer #1: Thank you for inviting me to review this manuscript. I have read the manuscript carefully, focusing on Method and Results. Although it is an important topic, please See the attached file fro your feedback to improve your manuscript.

Reviewer #2: The topic of this manuscript is important and show what really happen in reality especially in Indonesia setting. Mental health even it become one of the health priority in health ministry program, still the application especially in the community are still facing many challenges. Especially with lay workers or Kader-- they have significant role however, it’s only their side job, they don’t have well financial support and because it is voluntary, the Kader can be replaced over time, the same with nurses in community health centre, too many programs for one man job. I think, this paper should address more this aspect in discussion and also in future recomendation.

Reviewer #3: The manuscript addresses an important and timely topic: the acceptability of low-intensity psychological interventions in Indonesia, particularly in a context where depression and anxiety remain undertreated and where task-shifting through non-specialist workers may offer a scalable solution. The study is strengthened by its multi-stakeholder design, inclusion of patients, families, health workers, Kader, and community/religious leaders, and use of the Theoretical Framework of Acceptability. The sample is substantial for qualitative work, with 140 participants across 12 sites in Java .

However, several areas require strengthening before publication.

First, the manuscript needs a clearer statement of its analytical contribution. The current framing is largely descriptive. The authors should explain more explicitly what this study adds beyond identifying barriers such as stigma, low literacy, privacy concerns, and workload. The strongest potential contribution is not simply that LIPIs are “acceptable,” but that acceptability in Indonesia is conditional, socially negotiated, and shaped by trust, privacy, gendered burden, religious/community legitimacy, and the perceived role of Kader.

Second, the concept of “acceptability” needs sharper theorisation. The manuscript uses the TFA well, but the findings suggest that several domains overlap, especially ethicality, affective attitude, perceived effectiveness, and intervention coherence. The authors should clarify how they distinguished these constructs during coding and interpretation. Otherwise, the analysis risks becoming a thematic mapping exercise rather than a theoretically informed qualitative analysis.

Third, the paper should engage more critically with the limitations of using the TFA in LMIC and collectivist contexts. The discussion begins to do this, noting that TFA was developed largely from high-income country literature and may underrepresent LMIC conceptualisations of acceptability. This is one of the most interesting parts of the paper and should be elevated earlier and developed more strongly as a key theoretical contribution.

Fourth, the manuscript should avoid overclaiming cultural generalisability. Data were collected only in Java, while Indonesia is highly diverse in ethnicity, religion, language, geography, and health system access. The limitation is acknowledged, but the title and conclusion sometimes imply broader Indonesian applicability. The authors should consistently specify “Java, Indonesia” or explain why findings may plausibly inform wider Indonesian implementation.

Fifth, the role of Kader requires deeper critical analysis. The manuscript reports high trust in Kader in rural areas but concerns in urban areas around confidentiality, gossip, and skills. This rural–urban contrast is highly important and should be treated as a central finding rather than a secondary observation. It has direct implications for implementation models: Kader-led delivery may not be equally acceptable across settings.

Sixth, the gender dimension should be strengthened. The finding that women may need permission from husbands and face household responsibilities that limit participation is important. This should be linked more explicitly to gendered autonomy, household decision-making, and mental health service access. At present, gender appears in the findings but is underdeveloped analytically.

Seventh, the manuscript would benefit from a clearer implementation implications section. The findings point to several practical recommendations: community mental health literacy campaigns, confidentiality protocols for Kader, role clarification for religious/traditional healers, compensation or workload protection for Kader, flexible session timing, and privacy-sensitive delivery models. These should be presented in a concise table mapping barriers to adaptation strategies.

Finally, some writing needs tightening. Several sentences are long, repetitive, or grammatically awkward. The abstract and conclusion should be more direct. For example, rather than saying the study shows “cultural, structural, and interpersonal factors shape intervention acceptability,” the authors could state: “LIPI acceptability in Java depends on whether interventions can be made private, trusted, culturally legitimate, low-burden, and clearly differentiated from general counselling.”

Overall, this is a valuable and publishable manuscript, but it needs a stronger analytical argument, clearer theoretical contribution, and more concrete implementation guidance.

Reviewer #4: The paper entitled “Stakeholder perspectives on the acceptability of low-intensity psychological interventions in Indonesia: A theoretically framed qualitative study” addresses an important and timely topic in global mental health and implementation research. The manuscript is generally well written, methodologically sound, and draws on a large and diverse qualitative dataset. However, there are several areas that require clarification, tightening, and further conceptual refinement before the manuscript is suitable for publication. These are outlined below.

Abstract

• Page 2, Lines 24 to 25: The sentence “Low-intensity psychological interventions, delivered by trained non-specialists offer a promising pathway…” requires a comma after “non-specialists.”

• Page 2, Lines 36 to 42: The Results section is quite dense and introduces multiple TFA constructs simultaneously. Consider simplifying the wording and reducing conceptual repetition to improve readability.

• Page 3, Lines 44 to 48: The claim that this study is “one of the first theoretically framed qualitative explorations” may be somewhat overstated unless supported by clearer positioning within the existing literature. Please moderate this statement.

Introduction

• Page 4, Lines 52 to 64: The opening paragraph is overly dense and attempts to cover prevalence, economic burden, workforce shortages, and treatment gaps simultaneously. Consider breaking this into shorter and more focused paragraphs.

• Page 4, Lines 61 to 62: The statement that the ratio of therapists in LMICs is “approximately 0.5% that of high-income countries” is difficult to interpret as currently written. Please revise for clarity.

• Page 5, Lines 82 to 87: The discussion on low-intensity interventions and task shifting is useful. However, it would strengthen the paper to briefly distinguish low-intensity interventions from general counselling earlier in the manuscript, especially given that confusion around this issue later becomes a major finding.

• Page 6, Lines 100 to 109: The rationale for conducting this study in Indonesia is clear. However, the discussion on Muslim-majority settings, sociocultural norms, and implementation gaps could be better integrated conceptually rather than presented as separate issues.

Materials and Methods

• Page 7, Lines 125 to 126: The authors state that a qualitative descriptive design was used. Please provide a brief rationale for why this design was selected over other qualitative approaches such as phenomenology, grounded theory, or reflexive thematic analysis.

• Page 8, Lines 160 to 162: The manuscript states that data collection continued until “data saturation was achieved.” Please explain how saturation was operationalised and determined, particularly across the different stakeholder groups.

• Page 8, Lines 167 to 168: It is unclear how participants with anxiety and depression were identified. Please clarify whether these were clinically diagnosed conditions, self-reported experiences, screening-based identifications, or based on prior service contact.

• Page 9, Lines 184 to 188: The recruitment strategy for community stakeholders appears broad and heterogeneous. Please clarify whether any effort was made to ensure balance across stakeholder categories such as traditional healers, religious leaders, policymakers, and community leaders.

• Page 10, Lines 201 to 202: The sentence “focus group discussions last just between 60 and 70 minutes” requires grammatical revision.

• Page 10, Lines 211 to 212: Only 5% of transcripts were back translated. Please provide a brief justification for why this proportion was considered adequate for ensuring translation accuracy.

Data Analysis

• Page 15, Lines 278 to 293: The analysis section is generally strong. However, there appears to be some conceptual stretching of the TFA constructs. For example, “perceived need” is incorporated under affective attitude, and “collective ethicality” is introduced as an extension of the framework. Please clarify more explicitly which findings emerged inductively beyond the original TFA framework rather than retrospectively fitting all findings into TFA domains.

• Page 15, Lines 281 to 282: There is a typographical error in the phrase “ethicality and and agreeing.”

• Page 15, Lines 286 to 286: The statement regarding data “that fell out with the TFA framework” would benefit from further clarification. How were these inductive themes ultimately integrated into the analysis?

Results

• Page 16, Lines 313 to 322: The findings regarding mental health literacy and stigma are important. However, similar points recur repeatedly across later sections. The Results section would benefit from further condensation and synthesis to reduce repetition.

• Page 16, Lines 317 to 319: The explanation of local terminology relating to mental illness is useful, though the phrase “people with psychological problem who are risk of developing mental health disorders” requires grammatical revision.

• Page 17, Lines 338 to 343: The confusion between LIPIs and counselling appears to be one of the manuscript’s most important findings. It may be useful to expand the interpretation of this issue rather than treating it primarily as low intervention coherence.

• Page 18 to 22: Several quotations are quite long and occasionally difficult to follow due to translation structure. Some quotations could be shortened without losing conceptual meaning.

• Page 19, Lines 393 to 399: The discussion around self-reliance, autonomy, and social isolation is interesting and could be analysed more deeply in relation to Indonesian sociocultural norms.

• Page 21, Lines 443 to 450: The role of religious and traditional healers is highly relevant. However, the analysis remains somewhat descriptive. It would strengthen the paper to discuss more critically how traditional and biomedical understandings of mental distress intersect or potentially conflict.

• Page 22 to 24: The findings relating to stigma, privacy, and confidentiality are among the strongest parts of the manuscript. However, there is considerable overlap between discussions of ethicality, stigma, trust, and social judgement. Consider streamlining these sections conceptually.

• Page 25, Lines 529 to 534: The urban versus rural differences in trust toward Kader are particularly important findings. These distinctions deserve greater analytic attention in both the Results and Discussion sections.

• Page 26, Lines 549 to 589: The discussion of burden and opportunity costs is valuable, especially the gendered dimensions of participation. However, some sections become repetitive and could be tightened.

Discussion

• Page 28, Lines 600 to 609: The opening paragraph of the Discussion provides a good summary. However, several points raised here are repeated multiple times later in the section.

• Page 29, Lines 634 to 646: The discussion around affective attitude and cultural value alignment is interesting. However, the manuscript would benefit from a deeper explanation of how Islamic, communal, and gender norms specifically shape intervention acceptability.

• Page 30, Lines 648 to 660: The discussion around stigma and confidentiality is useful. However, the phrase “the modality of the intervention” conflicting with norms of discretion could be explained more clearly with practical examples.

• Page 31 to 32, Lines 681 to 702: This is arguably one of the strongest sections of the manuscript. The reflection on the applicability of TFA constructs in LMIC settings is thoughtful and theoretically important. Consider foregrounding some of these conceptual reflections earlier in the paper.

Strengths and Limitations

• Page 32 to 33, Lines 704 to 724: The limitations section is appropriately balanced and realistic. The acknowledgment that most participants were recruited through health services is particularly important.

• Page 33, Lines 719 to 724: The reflections on translation challenges are appreciated. It may be helpful to briefly indicate whether these challenges materially influenced interpretation of any themes.

Conclusion

• Page 33 to 34, Lines 735 to 742: The conclusion is generally appropriate and grounded in the findings. However, phrases such as “novel insights” and “contextually tailored, culturally congruent” are repeated frequently throughout the manuscript and could be simplified for conciseness.

Thank you.

**********

what does this mean?). If published, this will include your full peer review and any attached files.

If you choose “no”, your identity will remain anonymous but your review may still be made public.

**Do you want your identity to be public for this peer review?**  For information about this choice, including consent withdrawal, please see our Privacy Policy

Reviewer #1: No

Reviewer #2: **Yes:**  Hema Malini

Reviewer #3: No

Reviewer #4: **Yes:**  Prof. Franklin Glozah

**********

[NOTE: If reviewer comments were submitted as an attachment file, they will be attached to this email and accessible via the submission site. Please log into your account, locate the manuscript record, and check for the action link "View Attachments". If this link does not appear, there are no attachment files.]

To ensure your figures meet our technical requirements, please review our figure guidelines: https://journals.plos.org/plosone/s/figures

You may also use PLOS’s free figure tool, NAAS, to help you prepare publication quality figures: https://journals.plos.org/plosone/s/figures#loc-tools-for-figure-preparation.

NAAS will assess whether your figures meet our technical requirements by comparing each figure against our figure specifications.

Attachment Submitted filename: Review PLoS LIPI.docx

# Author comment: Author response to Decision Letter 1

**DOI:** 10.1371/journal.pone.0358869.r002

7 Aug 2026

See attachment.

Attachment Submitted filename: Response to reviewers PLOS Susanti Cover letter.docx

# Peer review: Decision Letter 1

**DOI:** 10.1371/journal.pone.0358869.r003

**Authors:** Kristiawan Indriyanto

18 Aug 2026

Dear Dr. Brooks,

Thank you for submitting your manuscript to PLOS One. After careful consideration, we feel that it has merit but does not fully meet PLOS One’s publication criteria as it currently stands. Therefore, we invite you to submit a revised version of the manuscript that addresses the points raised during the review process.

Please submit your revised manuscript by Oct 02 2026 11:59PM. If you will need more time than this to complete your revisions, please reply to this message or contact the journal office at plosone@plos.org. When you're ready to submit your revision, log on to https://www.editorialmanager.com/pone/ and select the 'Submissions Needing Revision' folder to locate your manuscript file.

- A letter that responds to each point raised by the academic editor and reviewer(s). You should upload this letter as a separate file labeled 'Response to Reviewers'.
- A marked-up copy of your manuscript that highlights changes made to the original version. You should upload this as a separate file labeled 'Revised Manuscript with Track Changes'.
- An unmarked version of your revised paper without tracked changes. You should upload this as a separate file labeled 'Manuscript'.

If you would like to make changes to your financial disclosure, please include your updated statement in your cover letter. Guidelines for resubmitting your figure files are available below the reviewer comments at the end of this letter.

If applicable, we recommend that you deposit your laboratory protocols in protocols.io to enhance the reproducibility of your results. Protocols.io assigns your protocol its own identifier (DOI) so that it can be cited independently in the future. For instructions see: https://journals.plos.org/plosone/s/submission-guidelines#loc-laboratory-protocols. Additionally, PLOS One offers an option for publishing peer-reviewed Lab Protocol articles, which describe protocols hosted on protocols.io. Read more information on sharing protocols at https://plos.org/protocols?utm_medium=editorial-email&utm_source=authorletters&utm_campaign=protocols.

As the corresponding author, your ORCID iD is verified in the submission system and will appear in the published article. PLOS supports the use of ORCID, and we encourage all coauthors to register for an ORCID iD and use it as well. Please encourage your coauthors to verify their ORCID iD within the submission system before final acceptance, as unverified ORCID iDs will not appear in the published article. *Only*  the individual author can complete the verification step; PLOS staff *cannot*  verify ORCID iDs on behalf of authors.

We look forward to receiving your revised manuscript.

Kind regards,

Kristiawan Indriyanto

Academic Editor

PLOS One

Journal Requirements:

If the reviewer comments include a recommendation to cite specific previously published works, please review and evaluate these publications to determine whether they are relevant and should be cited. There is no requirement to cite these works unless the editor has indicated otherwise.

[Note: HTML markup is below. Please do not edit.]

Reviewers' comments:

Reviewer’s Responses to Questions

**Comments to the Author**

Reviewer #3: (No Response)

Reviewer #4: All comments have been addressed

**********

2. Is the manuscript technically sound, and do the data support the conclusions?

Reviewer #3: No

Reviewer #4: Yes

**********

3. Has the statistical analysis been performed appropriately and rigorously?

Reviewer #3: No

Reviewer #4: N/A

**********

4. Have the authors made all data underlying the findings in their manuscript fully available?

The PLOS Data policy

Reviewer #3: No

Reviewer #4: Yes

**********

5. Is the manuscript presented in an intelligible fashion and written in standard English?

Reviewer #3: No

Reviewer #4: Yes

**********

Reviewer #3: Thank you for the opportunity to review this manuscript. The study addresses an important and timely topic in global mental health, particularly the potential role of low-intensity psychological interventions in improving access to care in resource-constrained settings. I appreciate the authors’ efforts in conducting a large multi-stakeholder qualitative study across several sites in Java. However, despite these strengths, I have several substantial conceptual and methodological concerns that, in my view, limit the validity and contribution of the current manuscript.

1. The study claims to assess “acceptability,” but participants had not actually experienced the intervention

This is the most fundamental concern. The study was conducted before implementation of the LIPI, specifically to inform cultural adaptation. Thus, participants were evaluating a proposed intervention model, rather than an intervention they had actually received or delivered. The authors themselves acknowledge elsewhere in the response that the intervention presented to participants was not yet a standardized protocol but a proposed guided self-help model of approximately 6–8 sessions delivered by Kader.

Consequently, much of what is described as “acceptability” is more accurately anticipated or hypothetical acceptability. This distinction is critical under the TFA because prospective perceptions may differ substantially from experienced acceptability once participants encounter the actual intervention burden, therapeutic relationship, confidentiality arrangements, Kader competence, session intensity, and treatment outcomes.

Yet the manuscript makes statements such as:

“LIPI acceptability in Java depends on whether interventions are perceived as private, trustworthy, culturally legitimate, and feasible...”

and discusses “potential for uptake.” Without actual intervention exposure, these conclusions are stronger than the design permits.

This is not readily fixed through textual revision. The central outcome in the title, objectives, analysis and conclusions is acceptability, while the empirical data primarily capture expectations about a hypothetical intervention.

2. There is a serious construct-validity problem because many participants did not clearly understand what a LIPI was

The manuscript identifies low intervention coherence as a major finding: participants frequently conflated guided self-help/LIPIs with counselling. This creates a circular methodological problem.

If participants did not adequately distinguish the intervention from counselling, it becomes unclear what intervention they were actually judging when answering questions about acceptability. Their positive attitudes, perceived effectiveness, burden, ethicality, or preferences may refer partly to counselling, general mental-health support, or simply receiving additional help—not specifically to the proposed LIPI.

The problem becomes particularly apparent when the authors report that participants expressed “hope, optimism, and acceptance” toward the intervention despite limited understanding of it.

This undermines the interpretability of several TFA domains. For example, it is difficult to establish meaningful perceived effectiveness for an intervention whose mechanism, intensity, provider role, and distinction from existing counselling were not consistently understood.

Low intervention coherence is presented as a substantive result, but it simultaneously threatens the validity of the entire acceptability assessment. The manuscript does not sufficiently separate “acceptability of receiving mental-health support” from “acceptability of this specific LIPI model.”

3. The sampling strategy creates substantial selection bias and limits the claimed community perspective

Recruitment relied on purposive and network-based sampling through primary-care services, community organisations and professional contacts, and response rates were not systematically recorded.

More importantly, individuals with anxiety/depression were identified through primary-care professionals rather than standardized diagnostic procedures. The manuscript acknowledges that:

“Patients were identified via primary care professionals as having depression and/or anxiety rather than formal diagnostic screening procedures.”

This is especially problematic for a study whose central themes include stigma, low mental-health literacy, delayed help-seeking, and barriers to accessing services. People already visible to health/community networks are likely systematically different from precisely those populations most affected by stigma, mistrust, social isolation, or avoidance of formal services.

The exclusion of individuals with recent/current suicide risk and some other conditions further means that the findings cannot readily represent people with more complex or severe needs.

The sampling mechanism potentially selects participants who are comparatively connected to health/community systems and receptive to intervention. This may partly explain the reported “broad consensus” and positive attitudes and creates an important threat to the central conclusions about community acceptability.

4. The TFA appears to structure rather than genuinely deepen the analysis, producing a largely descriptive study

Despite being presented as a “theoretically framed” study, the analytical contribution remains limited. Indeed, an earlier reviewer identified exactly this problem, describing the manuscript as “largely descriptive” and noting that barriers such as stigma, low literacy, privacy concerns, and workload needed a clearer analytical contribution.

The revision reframes the contribution as acceptability being “conditional and socially negotiated,” shaped by trust, privacy, gendered burden and community legitimacy. However, much of the analysis still maps relatively predictable findings onto TFA categories—intervention coherence, affective attitude, ethicality, burden, perceived effectiveness, etc.—rather than demonstrating how the empirical findings extend, challenge, or substantially refine the TFA.

There is also conceptual overlap among domains. For example, privacy and stigma influence ethicality, affective attitude and willingness to participate; trust affects perceived effectiveness and acceptability; and cultural/religious legitimacy cuts across several constructs. This raises the possibility that the framework has imposed categorical organization on phenomena that are inherently relational.

The authors themselves claim that the findings show the need for “contextual interpretation” of TFA in LMIC settings, but the manuscript does not develop this far enough into a theoretically distinctive model or proposition.

For a very large qualitative dataset involving 140 participants and 12 sites, the analytical return is surprisingly modest. The paper provides useful descriptive implementation information, but the theoretical contribution does not appear proportionate to the scale of the study or sufficiently novel to overcome the other design limitations.

5. Several implementation conclusions extend beyond what an acceptability study can establish

The manuscript repeatedly moves from participants' perceptions toward claims concerning implementation, fidelity, engagement, scalability and future uptake. For example, it argues that misunderstanding the intervention may lead to future deviations in delivery style and intensity and recommends training, supervision and monitoring to support fidelity.

Similarly, the discussion interprets perceived need as a “powerful motivator” capable of overriding low literacy and intervention-coherence barriers and suggests strong demand/openness across groups.

But no intervention was implemented, so the study did not observe actual:

uptake → engagement → adherence → fidelity → effectiveness → retention → sustainability.

Indeed, the authors explicitly acknowledge that feasibility and sustainability are outside this study and are being examined in later work packages. This makes some of the implementation-oriented interpretations premature.

This issue is particularly important given the authors' own finding that Kader already face heavy workloads and nurses anticipate difficulties providing supervision and training because of staff shortages. Positive hypothetical attitudes therefore cannot establish that the intervention will be acceptable when those constraints become real during delivery.

The conclusions occasionally cross the boundary between anticipated acceptability and evidence about actual implementation. Addressing this adequately would require post-implementation data rather than simply further discussion.

Reviewer #4: The authors have satisfactorily addressed my previous comments and concerns. I am happy with the revisions made, and I have no further comments.

**********

what does this mean?). If published, this will include your full peer review and any attached files.

If you choose “no”, your identity will remain anonymous but your review may still be made public.

**Do you want your identity to be public for this peer review?**  For information about this choice, including consent withdrawal, please see our Privacy Policy

Reviewer #3: No

Reviewer #4: No

**********

[NOTE: If reviewer comments were submitted as an attachment file, they will be attached to this email and accessible via the submission site. Please log into your account, locate the manuscript record, and check for the action link "View Attachments". If this link does not appear, there are no attachment files.]

To ensure your figures meet our technical requirements, please review our figure guidelines: https://journals.plos.org/plosone/s/figures

You may also use PLOS’s free figure tool, NAAS, to help you prepare publication quality figures: https://journals.plos.org/plosone/s/figures#loc-tools-for-figure-preparation.

NAAS will assess whether your figures meet our technical requirements by comparing each figure against our figure specifications.

# Author comment: Author response to Decision Letter 2

**DOI:** 10.1371/journal.pone.0358869.r004

21 Aug 2026

See attached response to reviewers doc.

Attachment Submitted filename: PLOS Revision 2 Response to Reviewer.docx

# Peer review: Decision Letter 2

**DOI:** 10.1371/journal.pone.0358869.r005

**Authors:** Kristiawan Indriyanto

7 Sep 2026

Stakeholder perspectives on the anticipated acceptability of low-intensity psychological interventions in Java, Indonesia: A theoretically framed qualitative study.

PONE-D-25-65667R2

Dear Dr. Brooks,

We’re pleased to inform you that your manuscript has been judged scientifically suitable for publication and will be formally accepted for publication once it meets all outstanding technical requirements.

Within one week, you’ll receive an e-mail detailing the required amendments. When these have been addressed, you’ll receive a formal acceptance letter and your manuscript will be scheduled for publication.

An invoice will be generated when your article is formally accepted. Please note, if your institution has a publishing partnership with PLOS and your article meets the relevant criteria, all or part of your publication costs will be covered. Please make sure your user information is up-to-date by logging into Editorial Manager at Editorial Manager® and clicking the ‘Update My Information' link at the top of the page. For questions related to billing, please contact billing support.

If your institution or institutions have a press office, please notify them about your upcoming paper to help maximize its impact. If they’ll be preparing press materials, please inform our press team as soon as possible -- no later than 48 hours after receiving the formal acceptance. Your manuscript will remain under strict press embargo until 2 pm Eastern Time on the date of publication. For more information, please contact onepress@plos.org.

Kind regards,

Kristiawan Indriyanto

Academic Editor

PLOS One

Additional Editor Comments (optional):

Reviewers' comments:

Reviewer’s Responses to Questions

**Comments to the Author**

Reviewer #3: All comments have been addressed

**********

2. Is the manuscript technically sound, and do the data support the conclusions?

Reviewer #3: Yes

**********

3. Has the statistical analysis been performed appropriately and rigorously?

Reviewer #3: Yes

**********

4. Have the authors made all data underlying the findings in their manuscript fully available?

The PLOS Data policy

Reviewer #3: Yes

**********

5. Is the manuscript presented in an intelligible fashion and written in standard English?

Reviewer #3: Yes

**********

Reviewer #3: Congratulations on your manuscript! This is a wonderful achievement and a testament to your hard work, dedication, and commitment to research. Completing and publishing a manuscript requires considerable effort, from developing the initial idea and conducting the research to carefully analyzing the findings and responding to reviewers’ comments. I hope this publication makes a meaningful contribution to the field and reaches a wide academic and professional audience. May this accomplishment open new opportunities for collaboration, inspire further research, and lead to many more successful publications in the future. Once again, congratulations on this well-deserved achievement and best wishes for your continued success.

**********

what does this mean?). If published, this will include your full peer review and any attached files.

If you choose “no”, your identity will remain anonymous but your review may still be made public.

**Do you want your identity to be public for this peer review?**  For information about this choice, including consent withdrawal, please see our Privacy Policy

Reviewer #3: No

**********

# Editor report: Acceptance letter

**DOI:** 10.1371/journal.pone.0358869.r006

**Authors:** Kristiawan Indriyanto

PONE-D-25-65667R2

PLOS One

Dear Dr. Brooks,

I'm pleased to inform you that your manuscript has been deemed suitable for publication in PLOS One. Congratulations! Your manuscript is now being handed over to our production team.

At this stage, our production department will prepare your paper for publication. This includes ensuring the following:

* All references, tables, and figures are properly cited

* All relevant supporting information is included in the manuscript submission,

* There are no issues that prevent the paper from being properly typeset

You will receive further instructions from the production team, including instructions on how to review your proof when it is ready. Please keep in mind that we are working through a large volume of accepted articles, so please give us a few days to review your paper and let you know the next and final steps.

Lastly, if your institution or institutions have a press office, please let them know about your upcoming paper now to help maximize its impact. If they'll be preparing press materials, please inform our press team within the next 48 hours. Your manuscript will remain under strict press embargo until 2 pm Eastern Time on the date of publication. For more information, please contact onepress@plos.org.

You will receive an invoice from PLOS for your publication fee after your manuscript has reached the completed accept phase. If you receive an email requesting payment before acceptance or for any other service, this may be a phishing scheme. Learn how to identify phishing emails and protect your accounts at https://explore.plos.org/phishing.

If we can help with anything else, please email us at customercare@plos.org.

Thank you for submitting your work to PLOS One and supporting open access.

Kind regards,

PLOS One Editorial Office Staff

on behalf of

Dr. Kristiawan Indriyanto

Academic Editor

PLOS One
